# CLIC, a tool for expanding biological pathways based on co-expression across thousands of datasets

**DOI:** 10.1371/journal.pcbi.1005653

**Published:** 2017-07-18

**Authors:** Yang Li, Alexis A. Jourdain, Sarah E. Calvo, Jun S. Liu, Vamsi K. Mootha

**Affiliations:** 1 Howard Hughes Medical Institute and Department of Molecular Biology and the Center for Genomic Medicine, Massachusetts General Hospital, Boston, MA, United States of America and Department of Systems Biology, Harvard Medical School, Boston, MA United States of America; 2 Department of Statistics, Harvard University, Cambridge, MA, United States of America; 3 Broad Institute, Cambridge, MA, United States of America; Johns Hopkins University, UNITED STATES

## Abstract

In recent years, there has been a huge rise in the number of publicly available transcriptional profiling datasets. These massive compendia comprise billions of measurements and provide a special opportunity to predict the function of unstudied genes based on co-expression to well-studied pathways. Such analyses can be very challenging, however, since biological pathways are modular and may exhibit co-expression only in specific contexts. To overcome these challenges we introduce CLIC, CLustering by Inferred Co-expression. CLIC accepts as input a pathway consisting of two or more genes. It then uses a Bayesian partition model to simultaneously partition the input gene set into coherent co-expressed modules (CEMs), while assigning the posterior probability for each dataset in support of each CEM. CLIC then expands each CEM by scanning the transcriptome for additional co-expressed genes, quantified by an integrated log-likelihood ratio (LLR) score weighted for each dataset. As a byproduct, CLIC automatically learns the conditions (datasets) within which a CEM is operative. We implemented CLIC using a compendium of 1774 mouse microarray datasets (28628 microarrays) or 1887 human microarray datasets (45158 microarrays). CLIC analysis reveals that of 910 canonical biological pathways, 30% consist of strongly co-expressed gene modules for which new members are predicted. For example, CLIC predicts a functional connection between protein C7orf55 (FMC1) and the mitochondrial ATP synthase complex that we have experimentally validated. CLIC is freely available at www.gene-clic.org. We anticipate that CLIC will be valuable both for revealing new components of biological pathways as well as the conditions in which they are active.

## Author summary

A major challenge in modern genomics research is to link the thousands of unstudied genes to the pathways and complexes within which they operate. A popular strategy to infer the function of an unstudied gene is to search for co-expressing genes of known function using a single transcriptional profiling dataset. Today, there are literally thousands of transcriptional profiling datasets, and a special opportunity lies in querying entire compendia for co-expression in order to more reliably expand pathway membership. Such analyses can be challenging, however, as pathways can be highly modular, and different datasets can conflict in terms of providing evidence of co-expression. To overcome these challenges, we introduce a tool called CLIC, CLustering by Inferred Co-expression. CLIC accepts a pathway of interest, simultaneously partitioning it into modules of genes that exhibit striking co-expression patterns while also learning the number of modules. It then expands each module with new members, based on an integrated weighted co-expression score across the datasets. Three key innovations within CLIC–partitioning, background correction, and integration–distinguish it from other methods. A side benefit of CLIC is that it spotlights the datasets that support the co-expression of a given co-expression module. Our software is freely available, and should be useful for identifying new genes in biological pathways while also identifying the datasets within which the pathways are active.

## Introduction

A major challenge in modern genomics is to predict the function of unstudied genes and to organize them into biologically meaningful pathways. While genome sequencing and annotation have revealed roughly 20,000 protein-coding human genes, a large fraction still do not have any known function. A fruitful strategy for predicting the function of unstudied genes relies on detecting co-expression with pathways of known function [1–8]. This “guilt by association” strategy, typically applied using a single large profiling dataset, has been widely useful across different organisms and now represents a routine method in modern genomics research. Many algorithms are available for spotlighting co-expressed genes in an individual transcriptome dataset [6, 9, 10].

In principle, the sensitivity and specificity of this approach can be boosted by searching for co-expression that is prevalent across many datasets. For example, some pathways may be expressed only in certain cell types or conditions, and searching across many datasets increases the likelihood for identifying experimental datasets in which a given pathway is expressed and varying. Observing co-expression across many experimental datasets can increase confidence that the co-variation is occurring for biologically interesting reasons and not for trivial or technical considerations. Hence, the co-expression method can benefit tremendously from examining not one but many transcriptional profiling datasets.

In recent years there has been an explosion in the number of freely available transcriptional profiling datasets in repositories such as Gene Expression Omnibus (GEO) [11, 12] and The Cancer Genome Atlas (TCGA) [13]. Data analytical tools in the early days of microarrays suffered from the “large *p* small *n* problem,” i.e., the number of “features” is much larger than the number of data points (samples). But today, there are more genome-wide transcriptional profiling datasets than human protein encoding genes. As of 2015, GEO housed >60,000 mRNA expression datasets corresponding to ~1.5 million microarrays and billions of individual gene expression measurements (S1 Fig). A tremendous opportunity lies in harnessing this data to reconstruct biological networks.

Performing co-expression analysis across many datasets poses many analytical challenges. For example, how does one weight evidence of co-expression from different datasets if they give conflicting information? Several methods, including early ones from our group [14], have been designed to tackle this challenge, including MEM [15], Expression Screening [14], WeGET [16], SEEK [17], COXPRESdb [18], and GeneFriends [19, 20]. MEM inputs a single input gene (rather than a gene set), performs co-expression on each dataset separately, and then uses Robust Rank Aggregation [21] to integrate across datasets. The other methods are capable of accepting as input a gene set and use different methods to weight datasets by co-expression of the query genes. Expression Screening weights datasets using a modified Kolmogorov-Smirnov statistic similar to the one used in Gene Set Enrichment Analysis [22]. WeGET assesses an input gene set’s co-expression across ~1000 multi-tissue datasets using the N100 statistic (fraction of query genes found among the top 100 genes with highest average correlation with the query genes) [9], and integrates across datasets using Robust Rank Aggregation [21]. SEEK uses a cross-validation algorithm to weight datasets and it uses a “hubbiness correction” to correct for the bias that some genes are generally correlated with all other genes. COXPRESdb calculates pairwise gene correlations across thousands of GEO datasets weighted by sample redundancy, then evaluates co-expression strength via a mutual rank statistic. To handle an input gene set, COXPRESdb’s CoExSearch analyzes each query gene separately then averages the mutual rank statistic. GeneFriends constructs gene pairwise co-expression maps with a similar approach as COXPRESdb on 4000 human and 4000 mouse RNA-seq samples. For an input gene set, GeneFriends ranks the candidate genes by the number of gene friends they have in the input gene set and their corresponding p-values, with “gene friends” defined as the top 5% co-expressed genes. WeGet, SEEK, COXPRESdb and GeneFriends all provide intuitive and fast web interfaces for analyzing input gene sets.

Several features limit the utility of existing multi-dataset methods. First, most existing methods assume the genes in the input query gene set represent one coherent co-expressed module–that is, they assume all the input genes are similarly pairwise correlated. However, biological pathways often contain modules each with distinct, context-dependent co-expression patterns (e.g. fatty acid metabolism modules active in different tissues and prandial states [23]). Second, many methods do not consider the background pattern of gene co-expression within a dataset. Non-specific co-expression can arise from technical factors (e.g. datasets with high gene-gene correlations due to poor normalization) and from biological factors (e.g. datasets that consist of microarrays from two distinct tissues such that nearly all pairs of genes co-vary). Third, most methods integrate evidence from different datasets using clever heuristic methods, which are not guided by a unified statistical model and may not be statistically optimal.

We expect that overcoming these technical limitations will improve the functional predictions from large gene expression compendia. Here, we tackle these existing limitations through the design of an overarching Bayesian statistical model and implementation of a Markov chain Monte Carlo (MCMC) inference algorithm called CLIC, CLustering by Inferred Co-expression. Three key innovations of CLIC are how it (i) corrects for background co-expression per dataset, (ii) partitions the input genes into co-expression modules, and (iii) integrates across different datasets. The Bayesian inference algorithm simultaneously identifies the co-expression modules and selects datasets in which those modules show high co-expression over background. In doing so, CLIC also spotlights the datasets that may be relevant for a pathway of interest. Hence, CLIC is useful both for expanding pathways with new genes while also identifying datasets in which a query pathway may be varying and hence “active”.

## Results

### CLIC overview

**Fig 1:**
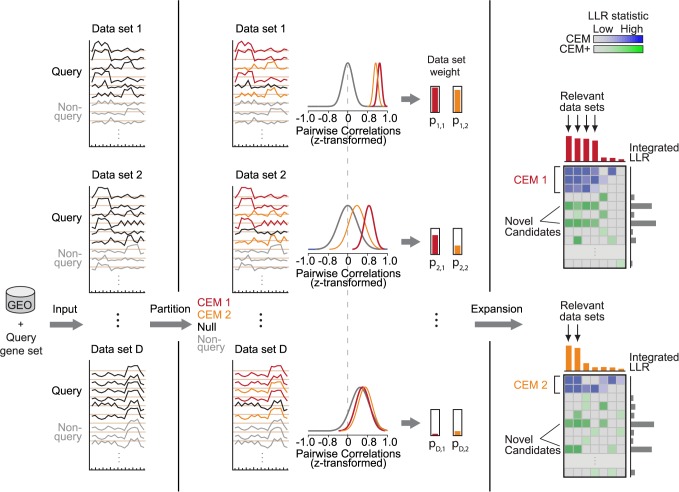
Schematic overview of CLIC. CLIC partitions an input Query gene set into co-expressed modules (CEMs), assigns weight to each dataset according to the intra-correlation of each module relative to background, and then predicts additional genes co-expressed with each CEM in high-weight datasets. CLIC inputs a compendium of *D* microarray data sets (e.g. from GEO) and an input Query gene set. In the Partition step, input genes are partitioned into distinct CEMs (in this example, CEM 1 in red, CEM 2 in orange), using a Bayesian partition model to simultaneously infer the number of CEMs and assign weights to datasets. Dataset weights quantify the significance of each intra-CEM correlation compared to the background distribution of correlation in each dataset (gray density curves). Genes from the input set that are not assigned to any CEM are assigned to a “Null” cluster. In the Expansion step, each CEM is expanded by identifying additional genes that show higher co-expression with the CEM genes compared to the gene-specific background distribution, scored by the log-likelihood ratio (LLR).

CLIC harnesses a compendium of gene expression datasets to partition an input gene set into disjoint co-expression modules (CEMs), highlights the most informative datasets for each CEM, and then expands each CEM with additional genes that frequently show specific co-expression across many datasets (Fig 1). CLIC accepts two user-defined inputs: (1) a compendium of *D* expression datasets (e.g. all GEO datasets from a single microarray platform) and (2) an input query gene set G (e.g. 44 genes in the proteasome complex). The CLIC algorithm consists of a Preprocessing step followed by Partition and Expansion steps. In the Partition step CLIC uses a Bayesian partition model, implemented via an MCMC sampler, to partition G into disjoint co-expression modules (CEMs), simultaneously learning the number of CEMs and assigning the posterior probability of selecting each dataset in support of each CEM. In the Expansion step, CLIC expands each CEM by scanning the transcriptome for co-expressed genes, quantified by an integrated log-likelihood ratio (LLR) score weighted for each dataset. The full details are provided in Methods, and briefly described below.

In the Preprocessing step, CLIC estimates two background distributions for each dataset in the compendium. For each dataset *d*, CLIC calculates a matrix of gene-gene correlations and applies Fisher’s *z*-transformation to this matrix so that the transformed correlations are approximately normally distributed (see Supplementary Materials). CLIC uses the transformed gene correlation matrix to calculate a *dataset-specific background distribution* with mean *θ*_*d*,0_ and variance $\sigma_{d,0}^{2}$. Next, for each gene *i* in dataset *d*, CLIC calculates a *gene-specific background distribution* with mean *θ*_*d*,0,*i*_ and variance $\sigma_{d,0,i}^{2}$ from all *z*-transformed correlations between gene *i* and all other genes in dataset *d*.

In the Partition step, CLIC partitions the input gene set G into *K* disjoint co-expressing modules (CEMs) (Fig 1) according to our Bayesian model, which assumes that genes within a CEM have similar and high (relative to the background) pairwise correlations within a supportive dataset in which the CEM is active and varying. CLIC employs an efficient MCMC sampling algorithm to search for the *maximum a posteriori* partitioning configuration of the input gene set G into *K* disjoint co-expressing modules (CEMs) (Fig 1), where *K* is simultaneously inferred from the data. For each CEM *k* and dataset *d*, CLIC calculates the dataset weight *p*_*d*,*k*_ that quantifies how strongly genes in CEM *k* co-express with each other compared to the dataset-specific background distribution (Fig 1). It is notable that these dataset weights spotlight relevant datasets in which the genes of CEM *k* are themselves co-expressed compared to the background. These weights are also used to score co-expressed genes in the Expansion step below. We note that not all input genes are assigned to a CEM. Singleton genes that co-express with the dataset-specific background distribution better than any CEM are assigned to a “null” group. Finally, each CEM is assigned a strength score, *ϕ*_*k*_, summarizing how well the genes in CEM *k* co-express with each other compared to the null model across the *D* datasets, using a weighted average of Bayes factors. In practice, we consider a CEM strength *ϕ*>0.1 to correspond to a module whose genes “co-express,” and CEM strength *ϕ*>1 as a module whose genes “strongly co-express.” The Partition step is essential to CLIC’s performance as the input gene set may not exhibit a single co-expressed module, but consist of distinct co-expressed modules.

In the Expansion step, for each CEM *k*, CLIC identifies additional genes (CEM_*k*_ +) that strongly co-express with the CEM genes across all datasets, where evidence from each dataset is weighted by how tightly the genes of the CEM themselves are co-expressed. For each CEM *k* and each candidate gene i∉G, CLIC calculates the log-likelihood ratio (LLR) to quantify gene *i*’s co-expression with CEM *k*. The LLR score in each dataset *d*, denoted as *LLR*_*k*,*i*,*d*_, is calculated between the foreground model H_1_ and background model H_0_. H_1_ assumes that the Fisher Z-transformed correlations between gene *i* and genes in CEM *k* follow the normal distribution with mean *θ*_*d*,*k*_ and variance $\sigma_{d,k}^{2}$ estimated from genes in CEM *k*. H_0_ assumes that correlations between gene *i* and genes in module *k* follow the gene-specific background normal distribution with mean *θ*_*d*,0,*i*_ and variance $\sigma_{d,0,i}^{2}$. The total integrated LLR score for a candidate gene *i* in CEM *k*, LLR_*k*,*i*_, is the summation of LLR scores over all the datasets weighted by the datasets weight *p*_*d*,*k*_ calculated in the Partition step (Fig 1). The CEM+ for each CEM is defined as the set of predictions with LLR scores exceeding a threshold, default 0. In practice, we consider LLR > 10 a good threshold to cutoff the significant CEM+ genes. The background H_0_ model is essential to the Expansion step, and is one feature that makes CLIC outperform traditional co-expression methods that do not take into account gene-specific background distributions. Since some genes are more generally correlated with other genes, these genes will always appear in the top of a CEM+’s prediction list trivially if we do not take into account its gene-specific background distribution. The LLR score, defined as the log-likelihood-ratio between foreground model and background model, serves as an integrated measure of co-expression.

### Implementation

We implemented CLIC in C++ and tested its performance on a list of input gene sets with various sizes. For an input gene set with ~50 genes across ~1800 transcriptional profiling datasets, the CLIC algorithm takes about 60 minutes on a standard Linux server using one single CPU. The computational time increases roughly linearly in the size of the input gene set, and also linearly in the number of transcriptional profiling datasets.

### Transcriptome compendia

We applied CLIC to two different compendia of mRNA datasets available from GEO. We selected the two most widely used mammalian platforms: the mouse Affymetrix chip Mouse430_v2 and the human Affymetrix chip HG-U133_Plus_2. For each platform, we downloaded all GEO datasets containing six or more microarray samples. We eliminated datasets with low quality and then re-normalized each dataset (see Methods). After filtering, we created a mouse compendium consisting of 1774 datasets (with 28628 Mouse430_v2 microarrays total) and a human compendium consisting of 1887 datasets (with 45158 HG-U133_Plus_2 microarrays total). Because we observed that the mouse compendium outperformed the human compendium on most known biological pathways (S2 Fig), we focused on the mouse analysis in the ensuing sections and discussion.

### Benchmarking the performance of CLIC

**Fig 2:**
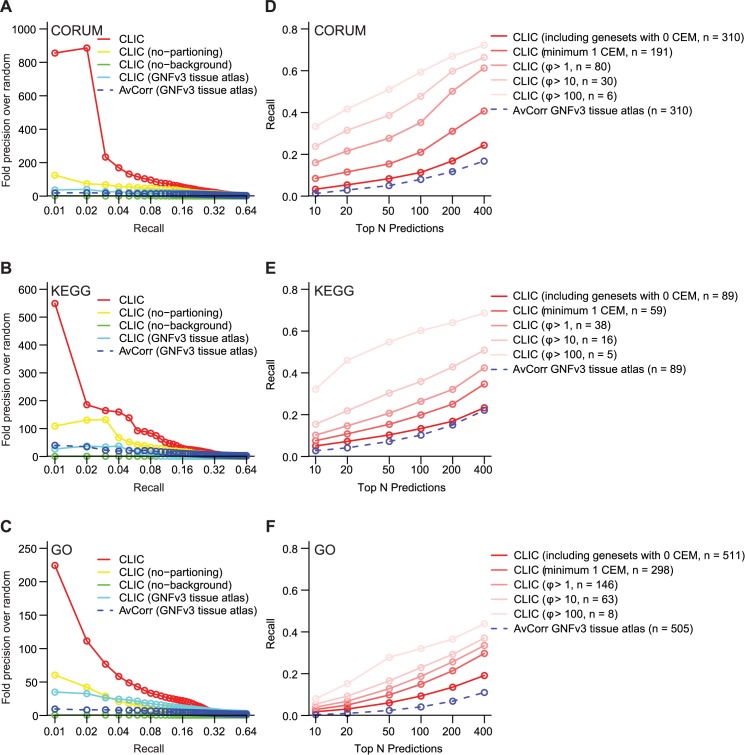
Benchmarking the performance of CLIC on three pathway databases. Leave-one-out cross-validation is shown for CORUM (A, D), KEGG (B, E) and GO (C, F) gene sets using 1774 mouse datasets from GEO. (A-C) Precision-recall curves show results based on CLIC and average correlation (AvCorr) using the GNFv3 tissue atlas. These plots highlight the utility of each of CLIC’s components: module-specific co-expression (CLIC vs CLIC no-partitioning), frequent co-expression (CLIC vs. CLIC GNFv3 tissue atlas), and specific co-expression (CLIC vs CLIC no-background). (D-F) Recall-rank curves show the recall (sensitivity) of different methods when looking at only top N predictions (N ranging 10–400). Results are shown for all gene sets, as well as for subsets with different CEM strength *ϕ* cut-offs, where n indicates the number of pathways used in generating the curves.

We assessed CLIC’s ability to recover known pathway genes using leave-one-out cross-validation (LOOCV) on curated pathways. We used three different databases of biological pathways and cellular complexes, considering all pathways containing 5–100 genes. We analyzed the curated databases separately since they contain some pathways in common. We utilized the CORUM database of protein complexes (310 complexes) [24], the KEGG database of metabolic pathways (89 pathways) [25], and the GO cellular component database from NCBI (511 gene sets) [26]. For each of the 910 annotated gene sets, we conducted LOOCV analysis and constructed precision-recall curves of CLIC’s performance over random chance–as has been used to assess similar algorithms [17] (see Methods) (Fig 2A–2C). Specifically, at each LLR threshold *t*, we calculated the precision (% of genes with LLR > *t* that are test genes) and recall (% of test genes with LLR > *t*). We also assessed specificity by measuring recall when only considering the top ranked predictions based on LLR (Fig 2D–2F).

LOOCV showed that CLIC could recover known pathway genes from these databases substantially better than random chance at all recall values (Fig 2A–2C). Considering just the top 50 predictions for each CEM from CORUM, the top 10% most co-expressed CEMs (*ϕ* > 10) have 40% recall (sensitivity) and the average CEM shows 10% recall (Fig 2D). Similar results are shown for KEGG (Fig 2E) and GO (Fig 2F). While not all input complexes and pathways are co-expressed, it is important to note that the CEMs with higher strength scores (*ϕ*) show correspondingly better recall, highlighting the value of CLIC’s measure of CEM strength as a measure of module co-expression (Fig 2D–2F).

Next we used LOOCV to compare CLIC to naive co-expression analysis within a single microarray dataset, the GNFv3 tissue atlas, which has been used widely for this purpose. Using this atlas, we computed the average correlation (AvCorr) of each gene *i*, defined as the mean Pearson correlation between gene *i* and all input genes in G. CLIC shows a significantly higher prediction accuracy than the simple average correlation using the GNFv3 tissue atlas (Fig 2). For example, considering just the top 50 predictions for each GO complex (Fig 2F), CLIC correctly predicted twice as many positive controls compared to AvCorr using GNFv3 tissue atlas. We note that there are other methods of co-expression analysis for a single dataset and in this comparison we simply intended to show the results of the simplest approach.

Finally, we used the LOOCV to assess just how important each of CLIC’s innovations–partitioning, background correction, and integration–are for its performance. Specifically, we evaluated how much of CLIC’s performance declines with (i) no partitioning of the input gene set, i.e. assuming that genes in input set G form a single CEM, (ii) no gene-specific background model (i.e., a gene is judged to be part of a module only by its likelihood of co-expression with members in the module regardless of its own co-expression tendency with other genes, that is the first term in the LLR definition), and (iii) no integration across datasets (*e*.*g*. using only the GNFv3 tissue atlas). As shown in Fig 2, compared with the full CLIC, “no-partitioning” and “GNFv3 tissue atlas” show substantially inferior performance and “no-background” shows almost no improvement over random chance. These analyses highlight the importance of partitioning, data integration, and especially background correction for the identification of co-expressed genes.

These LOOCV benchmark analyses highlight that CLIC can successfully predict functionally related genes of biological complexes/pathways with high specificity. As expected the method works best on the subset of biological pathways that are tightly co-expressed (Fig 2D–2F). Importantly, CLIC’s measure of CEM strength (*ϕ*) is a quantitative measure of the pathway module’s co-expression and indicates whether CLIC’s co-expression results are likely to be useful for a user’s gene set of interest. Similarly, CLIC produces a LLR score for each prediction that can inform the user how strongly each predicted gene is co-expressed with the input genes, compared to the background distribution. In Fig 2A–2C, CLIC’s predictions for different gene sets are merged by LLR scores, whereas in Fig 2D–2F, CLIC’s predictions are merged by rank. Comparing the relative performance of CLIC with AvCorr, it is shown that LLR score itself is much more informative than the ranks of genes in CEM+–highlighting the utility of the LLR score. In sum, cross-validation supports the utility of each part of CLIC’s framework.

### Comparing CLIC to other algorithms

**Fig 3:**
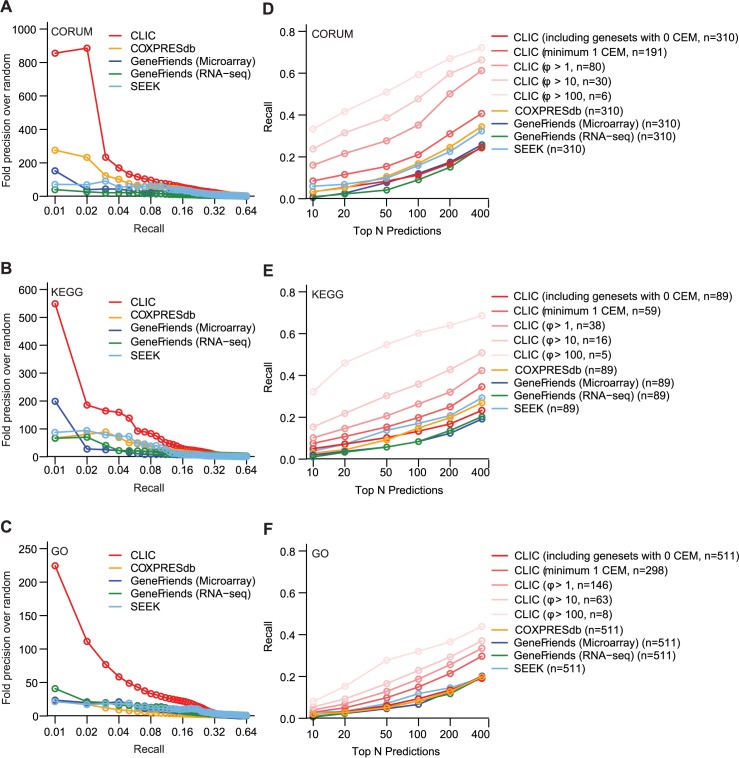
Comparing performance between CLIC and other co-expression algorithms. Leave-one-out cross-validation results for CLIC and other 3 methods (SEEK, COXPRESdb and GeneFriends with microarray and RNA-seq data) are shown as Precision-Recall curves (A-C) as well as Recall-Rank curves that show the recall (sensitivity) of algorithms when considering the top N predictions (D-F). Results are shown for CORUM (A,D), KEGG (B,E), and GO (C,F). n indicates the number of pathways used in generating each curve.

Next we systematically compared CLIC’s predictions to other co-expression algorithms using LOOCV on the 910 curated pathways (Fig 3). When considering the strongest predictions based on each tool’s prediction scoring metric, CLIC outperformed COXPRESdb [18], SEEK [17], and GeneFriends [20] (Fig 3A–3C). An alternative way to assess performance is to consider just the top ranked predictions, regardless of the tool’s scoring metric–although such rank-based analyses can conflate strong predictions with weak predictions arising from pathways that are poorly co-expressed. Based on LOOCV, all the algorithms showed fairly low recall within the top 100 predictions (5–15% recall), with COXPRESdb and SEEK outperforming CLIC on the CORUM and KEGG pathways (Fig 3D–3F). Unlike other algorithms, CLIC provides an explicit metric of module co-expression (CEM strength, *ϕ*), and CLIC shows substantially higher recall on the input pathways that are themselves strongly co-express–e.g. 30% recall within the top 100 predictions for the 80 CORUM complexes with *ϕ*>1 (Fig 3D). Taken together, we observe that CLIC offers two advantages: (1) it explicitly flags gene sets that are truly co-expressed using the CEM strength score, and (2) on these co-expressing pathways, CLIC provides high quality predictions.

### Application of CLIC to 910 canonical human complexes and pathways

**Fig 4:**
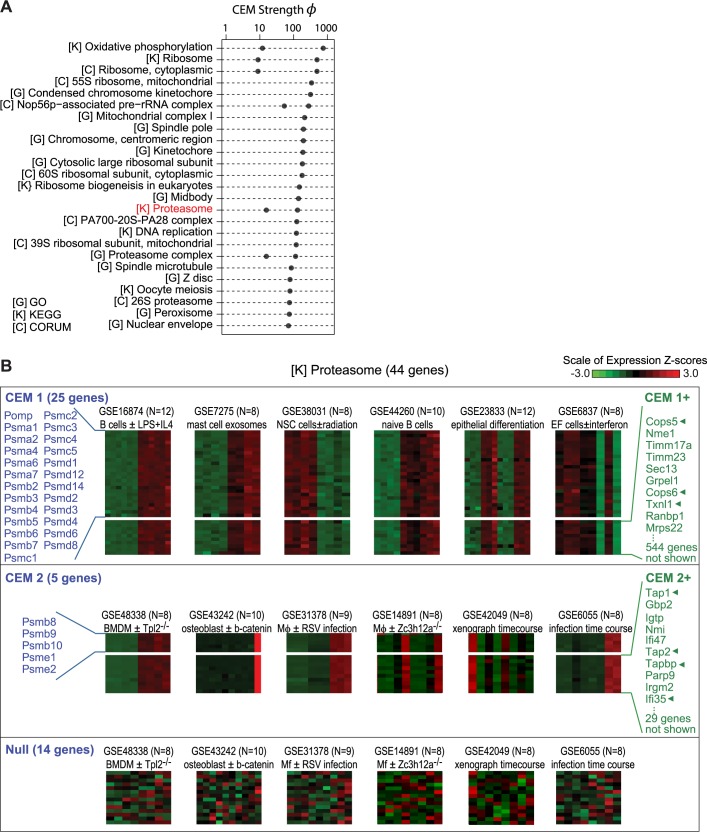
CLIC analysis of 910 canonical complexes and pathways. (A) Top 25 gene sets from CORUM [C], KEGG [K], or GO [G], ranked by strength of the top CEM. Red text indicates pathway detailed below. (B) CLIC results on the KEGG Proteasome show partitioning of 44 input genes into two CEMs (blue text) and 14 singletons. For each CEM, heat maps show expression profiles for the top 6 datasets (each row is one gene, each column is one sample, and cell color shows row-normalized z-scores across samples). For 14 null genes, the 6 datasets shown correspond to those in CEM2 –showing no co-expression across these sets. At the right, green text lists the top predictions in each CEM+, and arrowheads indicate predictions with recent experimental or human genetic support for functional association. Abbreviations: BMDM bone marrow derived macrophage; Mϕ macrophage.

We next sought to systematically identify which human pathways exhibit strong co-expression and could be expanded with new membership using CLIC. We assessed CLIC’s predictions from the 910 CORUM, KEGG and GO gene sets introduced above. We hypothesized that a subset of these human pathways will contain modules with genes that are frequently and specifically co-expressed. For such co-expression modules, CLIC can predict the function of uncharacterized genes for experimental validation. Overall, we found that 60% of the cellular components and pathways are co-expressed (defined as CEM strength *ϕ* > 0.1) and 29% are strongly co-expressed (CEM strength *ϕ* > 1). The pathways with the highest strength CEMs are summarized in Fig 4A, including Oxidative Phosphorylation (KEGG), Ribosome (KEGG), 55S ribosome mitochondrial (CORUM) and Condensed chromosome kinetochore (GO) (details shown in S3 Fig).

To illustrate the utility of CLIC, we show the co-expression modules and top RNA datasets for a high-scoring pathway: KEGG’s Proteasome pathway (Fig 4B). CLIC automatically partitions the 44 input genes into two co-expression modules with distinct co-expression patterns: CEM1 (25 genes, *ϕ* = 136.3) and CEM2 (5 genes, *ϕ* = 15.5), plus 14 singletons that did not cluster together (null group). Among the top predictions of CEM1 are three proteins known to interact with the proteasome based on existing literature: Txnl1 is a redox-active cofactor of the 26S proteasome [27] while Cops5 and Cops6 are subunits of the COP9 signalosome that function in the ubiquitin-proteasome pathway [28]. Interestingly, CEM2 contains 5 proteins (Psmb8, Psmb9, Psmb10, Psme1 and Psme2) that are known to function together in a specialized “immunoproteasome” involved in antigen presentation by the immune cells [29, 30]. Of note, 8 of the top 10 selected datasets for CEM2 involve infection/immune related experiments or cell types. Among the top predictions of CEM2 are Tap1, Tap2, and Tapbp–all associated with the TAP (transporter associated with antigen processing) complex that transports proteasome-generated peptides across the endoplasmic reticulum membrane prior to presentation on the cell membrane [31]. This example highlights that (i) a single biological gene set can consist of biologically relevant co-expression sub-modules, (ii) there are distinct datasets (tissues, cell-types, perturbations) in which different CEMs are co-expressed that are relevant to the underlying biology, and (iii) the top predictions include true biological associations. Thus, CLIC’s automatic clustering and expansion reveal insights into macromolecular complex organization and protein function.

### Predicting the function of poorly characterized human genes

**Fig 5:**
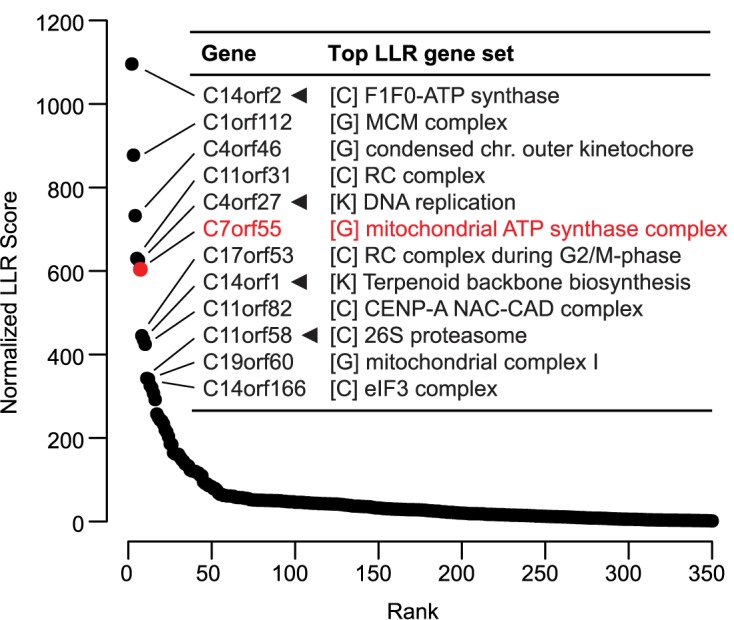
Functional predictions for uncharacterized human genes. 349 uncharacterized human genes (*X*-axis) are ranked by the highest normalized LLR score received from any of the 910 CORUM, KEGG, or GO annotated gene sets. The *y*-axis shows the top LLR score, normalized by the size of the corresponding gene set. Inset table shows the top predictions. Arrowheads indicate existing literature support of functional association, and red text indicates new experimental validation.

Next we aimed to systematically link genes of unknown function to one of the 910 curated pathways (Fig 5). We collected 349 human genes likely to have unknown function based on the NCBI gene name “C*N*orf*M*” indicating localization on chromosome *N* and open reading frame number *M*. We note some of these may have recently been defined functions not yet reflected in the name. CLIC is able to assign 349 human genes to 910 CORUM/KEGG/GO pathways. In particular, for each gene, we selected the CORUM, KEGG, or GO gene set that assigned the highest LLR prediction score, and predicted the gene is in that gene set with a normalized LLR score. Larger CEMs will naturally assign higher LLR scores to candidate genes, therefore to avoid this bias we defined a normalized LLR (nLLR) score as the original prediction LLR score divided by the size of the CEM. Among the top 10 predictions for these C*N*orf*M* genes (Fig 5, S1 Table), four are already supported by existing literature. First, CLIC’s prediction of *C14orf2* with the F_1_F_O_-ATP synthase is validated by studies that show *C14orf2* knock-down causes decreased ATP synthase levels and is possibly involved in the formation of ATP synthase dimers [32, 33]. Second, CLIC’s association between *C4orf27* and the DNA replication is consistent with a recent study showing *C4orf27* is a component of the DNA damage response [34]. Third, CLIC’s predicted association of *C14orf1* with KEGG pathways Terpenoid Backbone Biosynthesis and Steroid Biosynthesis is validated by experimental evidence linking the gene to sterol biosynthesis [35]. Fourth, CLIC’s prediction of *C11orf58* with 26S proteasome is supported by a large-scale experimental study showing the physical interaction between *C11orf58* and a proteasome component *Psmb7* [36].

### Experimental validation of *C7orf55*

**Fig 6:**
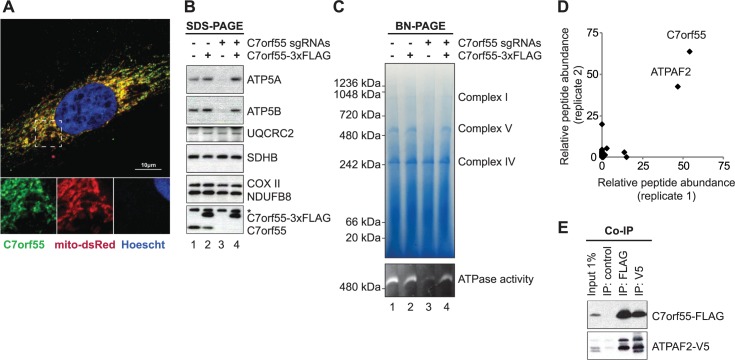
C7orf55 regulates ATP synthase activity. (A) Confocal microscopy of HeLa cells expressing a mitochondria-targeted version of dsRed (mito-dsRed) immunolabeled with antibodies to endogenous C7orf55. (B) Protein immunoblot analysis of K562 cells depleted for *C7orf55* and/or expressing a CRISPR-resistant version of *C7orf55*. * denotes an aspecific band recognized by the C7orf55 antibody. (C) Blue-native PAGE analysis on the cells described in (B) before (top) and after (bottom) in-gel ATPase activity reaction. (D) C7orf55-FLAG immunoprecipitation and mass spectrometry analysis of co-immunoprecipitated proteins from two replicates. (E) Co-immunoprecipitation of C7orf55-FLAG and ATPAF2-V5.

One of CLIC’s strongest novel predictions is the co-expression of the unstudied human gene *C7orf55* with mitochondrial ATP synthase complex (also known as complex V) (Fig 6). This gene product was reported to localize to the mitochondrion based on global mitochondrial proteomic surveys [37–39], however its function was uncharacterized. *C7orf55* was the 60^th^ most co-expressed gene with ATP synthase complex gene set (or 18^th^ after excluding OXPHOS subunits) based on CLIC, compared with much more distant ranks from other co-expression tools (S2 Table).

First, we confirmed mitochondrial localization (Fig 6A) by immunostaining with antibodies to endogenous C7orf55 and using confocal microscopy to observe co-localization with the mitochondrial compartment (visualized with Mito-dsRed).

Next we assessed the highly specific prediction that *C7orf55* is functionally related to complex V by (i) creating knockout cells and assessing the abundance and stability of all five OXPHOS complexes (Fig 6B and 6C) and (ii) by experimentally determining C7orf55’s binding partners (Fig 6D and 6E). We used CRISPR/Cas9 to knock out *C7orf55* in K562 cells (Fig 6B and 6C, column 3), and as a control to show specificity of the CRISPR knockout we overexpressed a CRISPR-resistant version of *C7orf55* (Fig 6B and 6C, column 4). In agreement with our prediction, in the absence of C7orf55 we observed a specific destabilization of the mitochondrial complex V using three assays: (i) the steady-state levels of the F_1_ ATP synthase subunits ATP5A and ATP5B were reduced in a denaturing SDS-PAGE gel (Fig 6B); (ii) the abundance of the fully-assembled complex V was reduced in a blue-native PAGE (Fig 6C, top panel); and (iii) the activity of complex V was reduced in an in-gel ATPase activity assay (Fig 6C, bottom panel). All the defects in complex V were entirely rescued by the reintroduction of a CRISPR-resistant version of *C7orf55* (Fig 6B and 6C, fourth column). Next, we overexpressed a tagged version of C7orf55 and assessed binding partners by using immunoprecipitation (using the FLAG tag) followed by mass spectrometry (Fig 6D). In two replicates, we observed only a single high-abundance endogenous binding partner: ATPAF2, a known assembly factor the F_1_ ATP synthase that is mutated in a human mitochondrial disease [40]. To confirm this binding association, we also tagged ATPAF2 with a V5 tag and showed that immunoprecipitation of ATPAF2-V5 binds C7orf55-FLAG (Fig 6E). These experiments confirm the validity of CLIC’s functional prediction that C7orf55 is required for mitochondrial ATP synthase function and specifically that C7orf55 binds a known assembly factor of this complex.

Since the preparation of this manuscript, human *C7orf55* was renamed *FMC1* based on the presence of the shared LYR protein domain with the yeast protein Fmc1p –however these short human and yeast proteins have no sequence homology detectable via BLASTP[41]. Yeast Fmc1p is required for stability of complex V in high temperature conditions [42], consistent with our experimental evidence for the human C7orf55. Furthermore, using genome-wide CRISPR screening we recently identified *C7orf55* as one of the 300 human genes required for oxidative phosphorylation, further validating our results, though this latter study did not assign to C7orf55 a specific role in complex V biology.

#### Software availability

CLIC is available via an online analysis portal (www.gene-clic.org) that enables users to login and launch analyses of their own gene sets containing as many as 250 genes. Results are emailed to users. In addition, the portal provides all software, source code, processed GEO datasets, and pre-computed analyses of 910 CORUM/KEGG/GO gene sets. We note that the online analysis requires a user login to run user jobs, jobs may take several hours to complete, and that for analysis of more than 250 genes users need to download the CLIC executables. We also note that CLIC requires an input gene set and cannot be run on a single gene query.

## Discussion

Here we introduced a Bayesian method, CLIC, for integrating across a large number of transcriptional profiling datasets to identify co-expression modules (CEMs) from an input gene set G, and to predict new genes showing frequent and specific co-expression with any CEM. CLIC is distinct from existing multi-dataset co-expression approaches in that (1) it is built on an overarching Bayesian hierarchical model that provides a statistically coherent algorithm to integrate many datasets for partitioning and expanding the gene set G; (2) it corrects for dataset-specific and gene-specific background distributions; (3) it automatically learns the number of CEMs; (4) it uses the LLR statistic as an integrated measure for co-expression across many datasets; and (5) it spotlights datasets in which a CEM is strongly co-expressing, hence identifying the datasets in which a pathway is potentially functioning.

While CLIC is more computationally intensive and slower than other similar methods, our benchmarking studies show that on the subset of pathways that are tightly co-expressed, CLIC provides more accurate results (Fig 3A–3C). However when considering the top ranked predictions of all pathways then SEEK and COXPRESdb showed slightly higher sensitivity (Fig 3D–3F).

CLIC is designed to operate on pathways that exhibit patterns of co-expression that are frequent (evidenced across many datasets) and specific (relative to background). Analysis of the 910 annotated pathways from three databases suggests that 60% of pathways have at least one co-expressed module (CEM strength *ϕ* > 0.1) and ~30% have a strongly co-expressed module (CEM strength *ϕ* > 1). The most strongly co-expressed cellular pathways include oxidative phosphorylation, cytosolic and mitochondrial ribosomes, kinetochores, spindle poles, proteasomes, and peroxisomes. We note that CLIC utilizes the co-expression within an input gene set as a “bait” with which to fish out relevant datasets from which new co-expressing members can be identified. As such it cannot operate on a single gene input. It is also not designed to operate on input pathways consisting of all singleton genes, i.e., it requires that the input pathway contains at least one pair of co-expressing genes.

We note that the CLIC inputs we showcased–the GEO compendium and the benchmark databases of curated pathways–each include potential sources of bias that will affect CLIC clustering and expansion results.

First, the two GEO database compendia we created contain a wide range of tissues and experimental perturbations, with certain tissues and cell lines over-represented. Naturally, CLIC will have increased power for pathways that vary in the tissues/conditions that are over-represented in these compendia. Changing the underlying compendia will change both the clustering and expansion of a user’s input gene set–as evidenced by better LOOCV performance of the 910 curated pathways on the mouse GEO compendia (Mouse430_v2) versus the human GEO compendia (1887 datasets on HG-U133_Plus_2) (Fig 2, S2 Fig). The mouse compendia may have shown better performance either for technical reasons (e.g. higher sensitivity/specificity of the microarray platform design) or for a wider variety of perturbations available from mouse tissues or cell lines. We observed that for some input gene sets such as the peroxisome, the main co-expression signature was obtained from expression across tissues and thus the large multi-tissue datasets swamped signal from highly interesting single-tissue datasets–thus our web-portal also contains GEO subsets excluding datasets with multiple tissues. In the future, other RNA expression compendia can be added, for example cancer-specific microarray datasets or additional platforms (such as from RNA-seq data).

Second, the three databases chosen to benchmark CLIC’s performance (CORUM, GO cellular components, KEGG metabolic pathways) contain substantial overlaps and are over-represented for protein complexes underlying translation and metabolism, and under-represented for signaling pathways. These benchmark databases were chosen for their high quality, and are not an exhaustive or representative set of all biological pathways. The high-quality CORUM database of protein complexes showed the best overall performance, suggesting that protein complexes may be more tightly co-expressed than KEGG metabolic pathways or GO cellular components (e.g. mitochondria, peroxisome) (Fig 2). While these benchmark databases demonstrate the ability of CLIC to make highly specific predictions, the chief utility of CLIC is for the analysis of a gene set of user’s interest.

The co-expression across conditions enables CLIC to predict specific functions for uncharacterized genes and to suggest links between well-studied pathways. We present strong functional predictions for hundreds of uncharacterized human genes (Fig 5 and S1 Table) including the link between *C7orf55* and complex V. Our *C7orf55* CRISPR knock-out experiments confirm C7orf55 protein is required for functional complex V, and provides new hypotheses into the potential assembly or regulation of this complex that we are actively exploring. Interestingly, CLIC highlights striking co-expression between the proteasome and two specific components of the mitochondrial import machinery (Timm71a and Timm23, Fig 3B). It is tempting to speculate that key components of the mitochondrial protein import machinery and the cytosolic proteasome are strongly co-expressed to guard against the toxic accumulation of proteins that fail to import into mitochondria [43, 44]. Together these examples highlight the utility of CLIC for providing specific hypotheses to elucidate function of unstudied proteins and of important regulatory connections between pathways.

While CLIC is designed to expand input pathways with new members, in practice, one of CLIC’s most useful features may be its ability to spotlight datasets or contexts that are likely to be of relevance for a pathway. While it is straightforward to scan across datasets to search for those in which a query gene set is simply highly expressed, CLIC helps spotlight those datasets in which the input genes are strongly varying and co-expressed over background—therefore more likely to be active and relevant. For example, our group recently identified the key components of the mitochondrial calcium uniporter [45–48], however we did not know which tissues and cellular contexts this channel was most physiologically relevant. Therefore we performed CLIC analysis on mitochondrial calcium uniporter components not to identify sub-modules or predict new components, but with the goal of identifying the existing datasets in which these genes had the most informative profiles. CLIC highlighted two datasets from mouse models of motor neuron disease (GSE5037, GSE5038) and skeletal muscle hypertrophy following over-expression of a transcriptional co-activator (GSE42473) [49]–thereby nominating physiological contexts within which the uniporter may be relevant. Similarly, CLIC analysis can be used to highlight the cell-lines best suited for designing experimental systems for functional characterization of a pathway or complex.

While co-expression across thousands of datasets will provide new insights into gene function, even more power can be gained by combining co-expression with complementary clues of protein function such as from protein interactions, co-occurrence of homologs within bacterial operons, or gene fusion events [50]. For mammalian genes we have found the most informative clues of protein function emerging from co-expression data in combination with phylogenetic profiling [45, 51, 52]. Indeed, given the utility of phylogenetic profiling we recently developed a Bayesian algorithm called CLIME (clustering by inferred models of evolution) to partition an input gene set into modules of co-evolving genes and then expand these modules with additional genes that have been lost together across evolution [53]. A key future challenge is to combine these methods (co-expression, co-phylogeny, protein interactions) in a principled manner to decipher pathway relationships amongst all human genes.

## Methods

### Formulation of the problem

CLIC takes two inputs: a query gene set G containing *n* genes, and a compendium of *D* gene expression datasets, where each dataset *d* is a matrix of gene expression values for *N* genes in the reference genome across multiple experimental samples. Let *r*_*d*,*i*,*j*_ denote the Pearson correlation between genes *i* and *j* in dataset *d*. To make the gene correlations approximately normally distributed, we apply Fisher’s *z*-transformation to each *r*_*d*,*i*,*j*_ so as to obtain the *z*-transformed correlation *z*_*d*,*i*,*j*_ (termed as the z-correlation henceforth):

$$z_{d,i,j}=\frac{1}{2}\ln\frac{1+r_{d,i,j}}{1-r_{d,i,j}}.$$

### Pre-processing: Inferring the background model

In the Pre-processing step, for each dataset *d*, CLIC estimates two background distributions directly from the data, both assumed to be normal. First, CLIC calculates the *dataset-specific background distribution* (mean *θ*_*d*,0_, variance $\sigma_{d,0}^{2}$) to model the dataset-specific z-correlation of all gene pairs:

$$\theta_{d,0}=\frac{2}{N(N-1)}\sum_{1\leq i<j\leq N}z_{d,i,j},\;\sigma_{d,0}^{2}=\frac{2}{N(N-1)}\sum_{1\leq i<j\leq N}{\left(z_{d,i,j}-\theta_{d,0}\right)}^{2}.$$

Next, for each gene *i*, CLIC calculates the *gene-specific background distribution* (with mean *θ*_*d*,0,*i*_, variance $\sigma_{d,0,i}^{2}$) to model the z-correlation between gene *i* and all other *N*−1 genes:

$$\theta_{d,0,i}=\frac{1}{N-1}\sum_{1\leq j\leq N,j\neq i}z_{d,i,j},\;\sigma_{d,0,i}^{2}=\frac{1}{N-1}\sum_{1\leq j\leq N,j\neq i}{\left(z_{d,i,j}-\theta_{d,0,i}\right)}^{2}.$$

Ideally, the *θ*_*d*,0_′s for unrelated gene pairs should be zero, but we choose to estimate these from the data to capture the heterogeneity between datasets as well as the random dataset-effect caused by the correlations among the sample correlations (Supplementary Materials). Since we have a huge amount of data to estimate *θ*_*d*,0_ and $\sigma_{d,0}^{2}$ (sample size is $\frac{1}{2}N(N-1)\approx 10^{8}$ for mouse and human genomes), their estimated values are sufficiently accurate so that throughout the article we treat *θ*_*d*,0_ and $\sigma_{d,0}^{2}$ as known parameters.

### Partitioning the input gene set into disjoint modules

The Partition step postulates a Bayesian partition model with automatic dataset selection, where both the partition and the selection indicators are inferred using MCMC. The goal is to partition the *n* genes in the input set G into *K* CEMs, indexed by *k* = 1,…,*K*, plus a null CEM indexed by *k* = 0. The number of CEMs *K* is unknown and estimated from the data. Let $Z_{d}={\left\{z_{d,i,j}\right\}}_{\forall i,j\in G}$ denote the matrix of pairwise z-correlations for genes in the input set G for dataset *d*. Let *J* = {*J*_*i*_: *i* = 1,…,*n*} index the CEM membership of each gene, where *J*_*i*_ = *k* indicates that gene *i* is in CEM *k*.

Only a subset of all datasets is selected for each CEM *k*. Let *S*_*d*,*k*_ ∈ {0,1} indicate whether dataset *d* is selected or not for CEM *k*. Let *S* = {*S*_1_,…,*S*_*D*_} and *S*_*d*_ = {*S*_*d*,1_,…,*S*_*d*,*K*_}. For a CEM *k* in a selected dataset *d* (i.e., *S*_*d*,*k*_ = 1), the within-CEM z-correlations are assumed to follow Normal distribution with mean *θ*_*d*,*k*_ and variance $\sigma_{d,k}^{2}$. The within-CEM z-correlations in an unselected dataset *d* (i.e., *S*_*d*,*k*_ = 0) and between-CEM z-correlations are assumed to follow Normal distribution with dataset-specific background model mean *θ*_*d*,0_ and variance $\sigma_{d,0}^{2}$.

CLIC makes the assumption that all genes in the same CEM *k* have intra-CEM z-correlations that are normally distributed and have the same mean *θ*_*d*,*k*_ and variance $\sigma_{d,k}^{2}$. Justifications for the assumption are given in the Supplementary Material. For genes in G not in the same CEM, the inter-CEM *z*-correlations are normally distributed and have background mean *θ*_*d*,0_ and variance $\sigma_{d,0}^{2}$. We denote this as follows. With a slight abuse of notation, we let *I*_*d*_ = {*I*_*d*,1_,…,*I*_*d*,*n*_} denote a function of *J* and *S*_*d*_, such that *I*_*d*,*i*_ = 0 if gene *i* is in a CEM *k* with *S*_*d*,*k*_ = 0 otherwise *I*_*d*,*i*_ = *J*_*i*_. We have

$$z_{d,i,j}|I_{d,i}=I_{d,j}=k∼N(\theta_{d,k},\sigma_{d,k}^{2}),\;k=1,\ldots ,K,$$

$$z_{d,i,j}|I_{d,i}\neq I_{d,j}\;or\;I_{d,i}I_{d,j}=0∼N(\theta_{d,0},\sigma_{d,0}^{2}),$$

where *N*(*θ*,*σ*^2^) denotes a normal distribution with mean *θ* and *σ*^2^.

Note that although the *z*_*d*,*i*,*j*_’s are correlated, we show in Supplementary Material that they can be approximated well by a random-effect Normal hierarchical structure. In other words, the *z*_*d*,*i*,*j*_’s can be viewed as being composed of one common random effect, specific to each dataset, plus an independent component. Hence, assuming that the covariance matrix for the *z*_*d*,*i*,*j*_’s is diagonal is a reasonable first-order approximation.

Conditional on *S*_*d*_, *θ*_*d*_ = {*θ*_*d*,0_,*θ*_*d*,1_,…,*θ*_*d*,*K*_} and *σ*_*d*_ = {*σ*_*d*,0_,*σ*_*d*,1_,…,*σ*_*d*,*K*_}, we have the following form of the likelihood function for *Z*_*d*_:

$$P(Z_{d}|\theta_{d},\sigma_{d},S_{d},I)=\underset{\mathrm{Null}\;\mathrm{Model}}{\underbrace{\left[\prod_{i<j:\;I_{d,i}\neq I_{d,j}}\frac{\text{exp}\{-\frac{{\left(z_{d,i,j}-\theta_{d,0}\right)}^{2}}{2\sigma_{d,0}^{2}}\}}{\sqrt{2\pi \sigma_{d,0}^{2}}}\right]}}∙\underset{\mathrm{Co-expression}\;\mathrm{Model}}{\underbrace{\prod_{k=1}^{K}\left[\prod_{i<j:\;I_{d,i}=I_{d,j}=k}\frac{\text{exp}\{-\frac{{\left(z_{d,i,j}-\theta_{d,k}\right)}^{2}}{2\sigma_{d,k}^{2}}\}}{\sqrt{2\pi \sigma_{d,k}^{2}}}\right]}},$$

where the product Π_*i*<*j*_ is over all pairs of 1 ≤ *i* < *j* ≤ *n*.

For each dataset *d*, we adopt conjugate Normal priors for *θ*_*d*,*k*_, *k* = 1, … *K*. Let constants *μ*_*θ*_ and *κ*_*θ*_ denote the prior mean and variance scale factor for the *θ*_*d*,*k*_’s,

$$\theta_{d,k}∼N(\mu_{\theta },\sigma_{d,k}^{2}/\kappa_{\theta }),\;k=1,\ldots ,K.$$

If CEM *k* is selected for dataset *d*, we assume high within-CEM z-correlations between genes in *k*, therefore it is natural to have *μ*_*θ*_ > 0. By default, we set *κ*_*θ*_ = 100 and *μ*_*θ*_ = 1.5, which corresponds to Pearson correlation ~ 0.9 and is roughly the average correlation among known co-expressed genes in oxidative phosphorylation gene sets in top 20 selected datasets. We also adopt conjugate inverse Gamma priors for the $\sigma_{d,k}^{2}$’s:

$$\sigma_{d,k}^{2}∼\text{Inv-Gamma}(\alpha_{\sigma },\beta_{\sigma }),\;k=1,\ldots ,K,$$

where *α*_*σ*_ and *β*_*σ*_ are hyper-parameters. By default we set *α*_*σ*_ = 1000 and *β*_*σ*_ = 1000. Fisher *z*-transformation is approximately variance-stabilizing so that *α*_*σ*_ and *β*_*σ*_ do not need to depend on mean *μ*_*θ*_.

We adopt simple Bernoulli priors for binary parameters *S*_*d*,*k*_’s,

$$P(S_{d,k}=1)=\pi_{S},\;d=1,\ldots ,D,k=1,\ldots ,K,$$

where *π*_*S*_ denotes the prior probability of a dataset being selected for a dataset *d*. Recall that the number of modules *K* is a function of module membership indicator *I*. To penalize the number of modules, the prior for indicator vector *I* is adopted as

$$P(I)\propto \text{exp}\{-v_{K}K\},$$

where *v*_*K*_ is a hyper-parameter to specify the intensity of penalization on the number of modules *K*. A larger *v*_*K*_ results in a smaller number of CEMs and more parsimonious model. By default, we set *π*_*S*_ = 0.1 and $v_{K}=\sqrt{n}D$.

Let *Z* = {*Z*_1_,…,*Z*_*D*_} denote the data for gene set G over the *D* datasets. Incorporating the prior and likelihood, we have the full posterior distribution as

$$\begin{array}{l} \;P\left(\theta ,\sigma ,S,I|Z\right)\\ \propto P\left(Z|\theta ,\sigma ,S,I\right)P\left(\theta \right)P\left(\sigma \right)P\left(S\right)P\left(I\right)\\ \propto \left[\prod_{d=1}^{D}P\left(Z_{d}|\theta_{d},\sigma_{d},S_{d},I\right)\right]\;\left[\prod_{d=1}^{D}P\left(\theta_{d}\right)\right]\;\left[\prod_{d=1}^{D}P\left(\sigma_{d}\right)\right]\;\left[\prod_{d=1}^{D}P\left(S_{d}\right)\right]\;P\left(I\right)\\ =\underset{\mathrm{Likelihood}\;\mathrm{Function}}{\underbrace{\prod_{d=1}^{D}\left\{\left[\prod_{i<j:\;I_{d,i}\neq I_{d,j}}\frac{\text{exp}\left\{-\frac{{\left(z_{d,i,j}-\theta_{d,0}\right)}^{2}}{2\sigma_{d,0}^{2}}\right\}}{\sqrt{2\pi \sigma_{d,0}^{2}}}\right]∙\prod_{k=1}^{K}\left[\prod_{i<j:\;I_{d,i}=I_{d,j}=k}\frac{\text{exp}\left\{-\frac{{\left(z_{d,i,j}-\theta_{d,k}\right)}^{2}}{2\sigma_{d,k}^{2}}\right\}}{\sqrt{2\pi \sigma_{d,k}^{2}}}\right]\right\}}}\\ \times \underset{\mathrm{Prior}\;\mathrm{distributions}\;\mathrm{for}\;\theta_{d,k}\;\text{and}\;\sigma_{d,k}^{2},\;d=1,\ldots ,D,\;k=1,\ldots ,K}{\underbrace{\prod_{d=1}^{D}\left\{\prod_{k=1}^{K}\left[\frac{1}{\sqrt{\frac{2\pi \sigma_{d,k}^{2}}{\kappa_{\theta }}}}\text{exp}\left\{-\frac{{\left(\theta_{d,k}-\mu_{\theta }\right)}^{2}}{\frac{2\sigma_{d,k}^{2}}{\kappa_{\theta }}}\right\}\right]∙\prod_{k=1}^{K}\left[\frac{\beta_{\sigma }^{\alpha_{\sigma }}}{\Gamma \left(\alpha_{\sigma }\right)}\sigma_{d,k}^{-2\left(\alpha_{\sigma }+1\right)}\text{exp}\left\{-\frac{\beta_{\sigma }}{\sigma_{d,k}^{2}}\right\}\right]\right\}}}\\ \times \underset{\mathrm{Prior}\;\mathrm{distribution}\;\mathrm{for}\;I}{\underbrace{\text{exp}\left\{-v_{K}K\right\}}}\times \underset{\mathrm{Prior}\;\mathrm{distribution}\;\mathrm{for}\;S}{\underbrace{\prod_{d=1}^{D}\prod_{k=1}^{K}{\left(\pi_{S}\right)}^{S_{d,k}}{\left(1-\pi_{S}\right)}^{1-S_{d,k}}}}. \end{array}$$ (1)

### Partition step implementation: Predictive updating and posterior sampling

In the Partition step, CLIC partitions the input set G into disjoint co-expressed modules (CEMs) that maximize the joint posterior probability of the partitioning configuration under our Bayesian model, simultaneously inferring the number of CEMs and each gene’s CEM membership. It is infeasible to enumerate all possible configurations of the posterior distribution in Eq (1) due to the large number of possible partitions and high dimensionality. Therefore, we apply Markov chain Monte Carlo (MCMC) [54] to draw samples from Eq (1). The conjugacy of prior distributions provides a nice analytical solution to the conditional distributions. We are able to sample from the posterior distribution by a canonical Gibbs sampler, iteratively updating variables by drawing from conditional distributions *P*(*θ*|*Z*,*σ*,*S*,*I*), *P*(*σ*|*Z*,*θ*,*S*,*I*) and *P*(*S*|*Z*,*θ*,*σ*,*I*). To update *I*, we (1) apply the idea of the collapsed Gibbs sampler to integrate out the nuisance parameters, which dramatically improves the sampling efficiency, and (2) run independent Markov chain with different *K*′s and retain the optimal $\hat{K}$ and $\hat{I}$ with the highest posterior probability.

In the Partition step, CEM membership indicator *I* and dataset selection indicator *S* are of our primary interest, and thus parameters *θ* and *σ* are nuisance parameters. We integrate out *θ* and *σ* to obtain the marginal likelihood function for *I* and *S*:

$$\begin{array}{l} \;P\left(Z|I,S\right)\\ =\int \int P\left(Z|\theta ,\sigma ,S,I\right)P\left(\theta \right)P\left(\sigma \right)d\theta d\sigma \\ =\prod_{d=1}^{D}\left[\prod_{k=1}^{K}{\left\{\frac{\left\{\frac{\beta_{\sigma }^{\alpha_{\sigma }}{\left(2\pi \right)}^{-\frac{C_{k}}{2}}}{\sqrt{1+\kappa_{\theta }^{-1}C_{k}}}\right\}\cdot \left\{\frac{\Gamma \left(\alpha_{\sigma }+\frac{C_{k}}{2}\right)}{\Gamma \left(\alpha_{\sigma }\right)}\right\}}{{\left(\beta_{\sigma }+\frac{1}{2}\left(\prod_{i<j:\;I_{d,i}=I_{d,j}=k}z_{d,i,j}^{2}+\kappa_{\theta }\mu_{\theta }^{2}-\frac{{\left(\kappa_{\theta }\mu_{\theta }+\prod_{i<j:\;I_{d,i}=I_{d,j}=k}z_{d,i,j}\right)}^{2}}{\kappa_{\theta }+C_{k}}\right)\right)}^{\alpha_{\sigma }+\frac{C_{k}}{2}}}\right\}}^{S_{d,k}}\right]\\ \times \prod_{d=1}^{D}\left[\prod_{i<j:\;I_{d,i}\neq I_{d,j}}\frac{\text{exp}\left\{-\frac{{\left(z_{d,i,j}-\theta_{d,0}\right)}^{2}}{2\sigma_{d,0}^{2}}\right\}}{\sqrt{2\pi \sigma_{d,0}^{2}}}\right], \end{array}$$

where *C*_*k*_ denotes the number of gene pairs in module *k*. Let *n*_*k*_ denote the number of genes in module *k*, then *C*_*k*_ = *n*_*k*_(*n*_*k*_−1)/2, *k* = 1,…,*K*.

We further integrate out *S* from likelihood function *P*(*Z*|*S*,*I*) and calculate the marginal likelihood of *I* using dynamic programming. This marginalization further improves the MCMC sampling efficiency.

$$\begin{array}{l} \;P\left(Z|I\right)\\ =\sum_{S_{1}\in {\left\{0,1\right\}}^{K}}P\left(S_{1}|I\right)\;\cdots \sum_{S_{D}\in {\left\{0,1\right\}}^{K}}P\left(S_{D}|I\right)\;P\left(Z|I,S\right)\\ =\sum_{S_{D}\in {\left\{0,1\right\}}^{K}}P\left(S_{D}|I\right)\left[\;\cdots \left[\sum_{S_{1}\in {\left\{0,1\right\}}^{K}}P\left(S_{1}|I\right)\;P\left(Z|I,S\right)\right]\right]\\ =\prod_{d=1}^{D}[\prod_{k=1}^{K}[\left(\pi_{S}\right)\left(\frac{\left\{\frac{\beta_{\sigma }^{\alpha_{\sigma }}{\left(2\pi \right)}^{-\frac{C_{k}}{2}}}{\sqrt{1+\kappa_{\theta }^{-1}C_{k}}}\right\}\cdot \left\{\frac{\Gamma \left(\alpha_{\sigma }+\frac{C_{k}}{2}\right)}{\Gamma \left(\alpha_{\sigma }\right)}\right\}}{{\left(\beta_{\sigma }+\frac{1}{2}\left(\prod_{i<j:\;I_{d,i}=I_{d,j}=k}z_{d,i,j}^{2}+\kappa_{\theta }\mu_{\theta }^{2}-\frac{{\left(\kappa_{\theta }\mu_{\theta }+\prod_{i<j:\;I_{d,i}=I_{d,j}=k}z_{d,i,j}\right)}^{2}}{\kappa_{\theta }+C_{k}}\right)\right)}^{\alpha_{\sigma }+\frac{C_{k}}{2}}}\right)\\ +\;\left(1-\pi_{S}\right)\left(\prod_{i<j:\;I_{d,i}=I_{d,j}=k}\frac{\text{exp}\left\{-\frac{{\left(z_{d,i,j}-\theta_{d,0}\right)}^{2}}{2\sigma_{d,0}^{2}}\right\}}{\sqrt{2\pi \sigma_{d,0}^{2}}}\right)]\cdot \left(\prod_{i<j:\;I_{d,i}\neq I_{d,j}}\frac{\text{exp}\left\{-\frac{{\left(z_{d,i,j}-\theta_{d,0}\right)}^{2}}{2\sigma_{d,0}^{2}}\right\}}{\sqrt{2\pi \sigma_{d,0}^{2}}}\right)]. \end{array}$$

The posterior distribution for *I* after integrating out *S*, *θ* and *σ* is

P(I|Z)∝P(I)P(Z|I).

For each *K* in {1,…,K}, we fix the number of CEMs to *K* and construct a Markov chain to traverse the space of all possible *I* with the stationary distribution being the target posterior distribution *P*(*I*|*Z*). K is the upper limit of *K* and by default we set K=5, which is enough for a medium-size canonical functional gene set that is supposed to consist of a limited number of CEMs. For an input gene set G with an extraordinarily large *n*, we may further increase *K* by schemes such as $K=\sqrt{n}$. We initialize the Markov chains with all genes being assigned to the null module, and then iterate the following updates for *M* = 1000 steps. In each iteration, for each gene *i* in turn, *i* = 1,…,*n*, we draw *I*_*i*_ from the conditional distribution *P*(*I*_*i*_|*Z*,*I*_[−*i*]_). In particular, we calculate *p*_*k*_ = *P*(*I*_*i*_ = *k*|*Z*,*I*_[−*i*]_) for *k* = 0,1,…,*K* and assign gene *i* into module *k* (null model is represented by *k* = 0) with probability *p*_*k*_. Each probability *p*_*k*_ is calculated as

$$\begin{array}{l} P(I_{i}=k|Z,I_{\left[-i\right]})=\frac{P(Z|I_{\left[-i\right]},I_{i}=k)P(I_{\left[-i\right]},I_{i}=k)}{\sum_{l=1}^{K}P(Z|I_{\left[-i\right]},I_{i}=l)\;P(I_{\left[-i\right]},I_{i}=l)}\\ \;=\frac{P(Z|I_{\left[-i\right]},I_{i}=k)}{\sum_{l=1}^{K}P(Z|I_{\left[-i\right]},I_{i}=l)}. \end{array}$$

### Point estimators

Let $\hat{K}$ and $\hat{I}$ denote the maximum a posterior (MAP) estimators for *K* and *I*. For each K∈{1,…,K}, let $I_{\left(K\right)}^{\left(1\right)},\ldots ,I_{\left(K\right)}^{\left(M\right)}$ denote the MCMC samples of *I* with the number of CEMs setting at *K*. We define $\hat{K}$ and $\hat{I}$ as the MCMC sample that maximizes the posterior probability

$$\hat{K},\hat{I}=\underset{K,I_{\left(K\right)}^{\left(m\right)}:\;K\in \{1,\ldots ,K\},m=1,\ldots ,M}{\arg\;\max}P(I_{\left(K\right)}^{\left(m\right)}|Z).$$

In the MCMC sampling, we integrated out parameters *θ*’s and *σ*’s and implemented the collapsed Gibbs sampler to improve the sampling efficiency. Once the partitioning is done and $\hat{I}$ is determined, CLIC estimates *θ*’s and *σ*’s by calculating their maximum likelihood estimates (MLEs) conditional on $\hat{I}$. Let ${\hat{\theta }}_{d,k}$ and ${\hat{\sigma }}_{d,k}^{2}$ denote the MLEs of *θ*_*d*,*k*_ and $\sigma_{d,k}^{2}$, then for *d* = 1,…,*D* and $k=1,\ldots ,\hat{K}$,

$$\begin{array}{l} {\hat{\theta }}_{d,k}=\frac{\sum_{i<j}I\{{\hat{I}}_{i}={\hat{I}}_{j}=k\}∙z_{d,i,j}}{\sum_{i<j}I\{{\hat{I}}_{i}={\hat{I}}_{j}=k\}}.\\ {\hat{\sigma }}_{d,k}^{2}=\frac{\sum_{i<j}I\{{\hat{I}}_{i}={\hat{I}}_{j}=k\}∙{\left(z_{d,i,j}-{\hat{\theta }}_{d,k}\right)}^{2}}{\sum_{i<j}I\{{\hat{I}}_{i}={\hat{I}}_{j}=k\}}. \end{array}$$

These estimates will be used in the Expansion step to predict the expanded list of genes for each CEM (denoted as CEM+).

### CEM strength measurement

For each CEM *k*, CLIC calculates a CEM strength, *ϕ*_*k*_, summarizing how well the genes in CEM *k* co-express with each other compared to the null model across the *D* datasets, using a weighted average of the Bayes factors [55]. For dataset *d*, the Bayes factor is calculated between the foreground (pairwise z-correlations for genes in CEM *k* share the same mean *θ*_*d*,*k*_ and variance $\sigma_{d,k}^{2}$) and the background model (pairwise z-correlations have background distribution mean *θ*_*d*,0_ and variance $\sigma_{d,0}^{2}$).

Let $Z_{d,k}=\{z_{d,i,j}:\;\forall i,j,\;1\leq i<j\leq n,\;{\hat{I}}_{d,i}={\hat{I}}_{d,j}=k\}$, and let *ϕ*_*k*_ denote the average Bayes factors over the *D* datasets, weighted by the probability that CEM *k* is selected for each dataset:

$$\phi_{k}=\frac{1}{D}\sum_{d=1}^{D}{\hat{p}}_{d,k}[\ln P(Z_{d,k}|\hat{I},S_{d,k}=1)-\ln P(Z_{d,k}|\hat{I},S_{d,k}=0)],$$

where

$${\hat{p}}_{d,k}=P(S_{d,k}=1|Z_{d,k},\hat{I})=\frac{\pi_{S}∙P(Z_{d,k}|\hat{I},S_{d,k}=1)}{\pi_{S}∙P(Z_{d,k}|\hat{I},S_{d,k}=1)+(1-\pi_{S})∙P(Z_{d,k}|\hat{I},S_{d,k}=0)},$$ (2)

and $P(Z_{d,k}|\hat{I},S_{d,k}=1)$ as well as $P(Z_{d,k}|\hat{I},S_{d,k}=0)$ are calculated as follows:

$$\begin{array}{l} \;P\left(Z_{d,k}|\hat{I},S_{d,k}=1\right)\\ =\int P\left(Z_{d,k}|\hat{I},\theta_{d,k},\sigma_{d,k}^{2},S_{d,k}=1\right)P\left(\theta_{d,k}\right)P\left(\sigma_{d,k}^{2}\right)d\theta_{d,k}d\sigma_{d,k}^{2}\\ =\frac{\left\{\frac{\beta_{\sigma }^{\alpha_{\sigma }}{\left(2\pi \right)}^{-\frac{C_{k}}{2}}}{\sqrt{1+\kappa_{\theta }^{-1}C_{k}}}\right\}\cdot \left\{\frac{\Gamma \left(\alpha_{\sigma }+\frac{C_{k}}{2}\right)}{\Gamma \left(\alpha_{\sigma }\right)}\right\}}{{\left(\beta_{\sigma }+\frac{1}{2}\left(\prod_{i<j:\;I_{d,i}=I_{d,j}=k}z_{d,i,j}^{2}+\kappa_{\theta }\mu_{\theta }^{2}-\frac{{\left(\kappa_{\theta }\mu_{\theta }+\prod_{i<j:\;I_{d,i}=I_{d,j}=k}z_{d,i,j}\right)}^{2}}{\kappa_{\theta }+C_{k}}\right)\right)}^{\alpha_{\sigma }+\frac{C_{k}}{2}}},\\ \;P\left(Z_{d,k}|\hat{I},S_{d,k}=0\right)\\ =\prod_{i<j:\;I_{d,i}=I_{d,j}=k}\frac{\text{exp}\left\{-\frac{{\left(z_{d,i,j}-\theta_{d,0}\right)}^{2}}{2\sigma_{d,0}^{2}}\right\}}{\sqrt{2\pi \sigma_{d,0}^{2}}}. \end{array}$$

A high CEM strength *ϕ*_*k*_ indicates that the genes in CEM *k* are frequently and specifically co-expressed across a large number of datasets.

### Expansion step: Calculation of log-likelihood ratio (LLR)

For each CEM *k*, CLIC scores the co-expression of each gene *i* not in G using the log-likelihood ratio (LLR) to compare the foreground model calculated from genes in CEM *k* (mean *θ*_*d*,*k*_ and variance $\sigma_{d,k}^{2}$) and the background model estimated from the preprocessing step (mean *θ*_*d*,0,*i*_ and variance $\sigma_{d,0,i}^{2}$). LLR_*k*,*i*,*d*_ is defined as

$$\begin{array}{l} {\text{LLR}}_{k,i,d}=\ln\frac{\prod_{j:{\hat{I}}_{j}=k}N(z_{d,j,i}|\theta_{d,k},\sigma_{d,k}^{2})}{\prod_{j:{\hat{I}}_{j}=k}N(z_{d,j,i}|\theta_{d,0,i},\sigma_{d,0,i}^{2})}\\ \;=\sum_{j:{\hat{I}}_{j}=k}\ln N(z_{d,j,i}|\theta_{d,k},\sigma_{d,k}^{2})-\ln N(z_{d,j,i}|\theta_{d,0,i},\sigma_{d,0,i}^{2}), \end{array}$$

where *N*(∙|∙,∙) denotes the normal distribution density function. The total integrated LLR score for a candidate gene *i* in CEM *k* is defined as the summation of individual LLR scores over the selected datasets with *S*_*d*,*k*_ = 1:

$${\text{LLR}}_{k,i}=\sum_{d=1}^{D}S_{d,k}[\sum_{j:{\hat{I}}_{j}=k}\ln N(z_{d,j,i}|\theta_{d,k},\sigma_{d,k}^{2})-\ln N(z_{d,j,i}|\theta_{d,0,i},\sigma_{d,0,i}^{2})].$$

Note that the LLR score is a function of true parameters *θ*, *σ*, *I* and *S*. We plug in the ${\hat{\theta }}_{d,k}$’s, ${\hat{\sigma }}_{d,k}^{2}$’s and $\hat{I}$ as estimates for the *θ*_*d*,*k*_’s, $\sigma_{d,k}^{2}$’s and *I*, and plug in ${\hat{p}}_{d,k}=P(S_{d,k}=1|Z_{d,k},\hat{I})$ calculated in Eq (2) as the conditional posterior mean estimator of *S*_*d*,*k*_. The estimated LLR is

$${\hat{\text{LLR}}}_{k,i}=\sum_{d=1}^{D}{\hat{p}}_{d,k}[\sum_{j:{\hat{I}}_{j}=k}\ln N(z_{d,j,i}|{\hat{\theta }}_{d,k},{\hat{\sigma }}_{d,k}^{2})-\ln N(z_{d,j,i}|\theta_{d,0,i},\sigma_{d,0,i}^{2})].$$

${\hat{\text{LLR}}}_{k,i}$ is the summation of estimated LLR scores over the *D* datasets, weighted by the posterior probability that CEM *k* is selected for each dataset *d*. For notational simplicity, we use LLR_*k*,*i*_ to denote ${\hat{\text{LLR}}}_{k,i}$.

### Compendia of transcriptional profiling datasets

We downloaded all mRNA expression microarray datasets from Gene Expression Omnibus (www.ncbi.nlm.nih.gov/geo/, 08/2014) associated with Affymetrix platform Mouse430_v2 (3,037 datasets) and HG-U133_Plus_2 (3,345 datasets). Using the Affymetrix probeset annotation tables downloaded 08/2014, we mapped Affymetrix probesets to NCBI Entrez identifiers; in cases where multiple probesets were mapped to one gene, we retained only the probeset with the lowest possibility for cross-hybridization (preferring probeset suffixes “at” over “a_at” over “s_at” over “x_at”). We performed quality control as follows: we (1) removed duplicated datasets and those that were subsets of other datasets based on GEO sample identifiers, (2) removed datasets with < 6 samples, since the estimation of correlation coefficient in small size datasets is usually not reliable, (3) identified all datasets in log-scale (maximum expression < 30) and re-scaled them (exponentiating them with base 2), (4) removed datasets with max expression < 1000. We normalized each dataset by scaling each sample column to have the same mean. Next we assessed the background distribution of all gene-gene correlations (*z*-transformed) to exclude datasets with low quality (e.g. small sample size or bad sample normalization) as follows. High quality sets show normal background distributions with small variance, whereas low quality sets show background distributions with multiple modes and large variance (S4 Fig). For each dataset *d*, we calculated the total variation distance *δ*(*p*_*d*_,*q*_*d*_) between the kernel fit of the background distribution, *p*_*d*_(*z*), and the normal fit of the background distribution, *q*_*d*_(*z*), as follows:

$$\delta (p_{d},q_{d})=\int_{-\infty }^{+\infty }|p_{d}(z)-q_{d}(z)|dz.$$

We removed a dataset *d* if *δ*(*p*_*d*_,*q*_*d*_) > 0.1 or $\sigma_{d,0}^{2}>1$.

### Benchmarking databases and algorithms

The CORUM database release 17.02.2012 was downloaded from Comprehensive Resource of Mammalian Protein Complex (downloaded 10/2015). KEGG metabolic and signaling pathways for human were downloaded from the KEGG Pathway Database, Release 58 [25], excluding 3 large terms ("Human Diseases", "Organismal Systems", "Environmental Response and Signaling") and excluding all genes that were present in greater than 3 different pathways. GO (Gene ontology) gene sets for cellular compartments were downloaded from the NCBI Gene database (*H*. *sapiens* genes, downloaded 12/2012). For experiments on GPL1261 datasets, human genes were mapped to mouse homologs via NCBI Homologene (downloaded 05/2014). To compare CLIC to other co-expression algorithms, we downloaded pre-computed results online (COXPRESdb [18]) or used online web portals (SEEK [17], GeneFriends [20]) to run each tool against the CORUM, KEGG, and GO curated databases using LOOCV as described below. For COXPRESdb, we downloaded the co-expression neighbors for each mouse gene (http://coxpresdb.jp/download.shtml), then reimplemented to CoExSearch procedure for using an input gene set as described (http://coxpresdb.jp/top_search.shtml#CoExSearch), in order to compute LOOCV for each of the 910 input pathways. We note that these three tools each use different transcriptional compendia, as described in each method.

#### Leave-one-out cross-validation

We conducted leave-one-out cross-validation (LOOCV) analysis to benchmark the performance of CLIC. For each input gene set G with *n* genes, we ran CLIC *n* times, in each case holding out gene *i* from the input set, then assessed whether gene *i* was predicted in any of the CEM+ expansions at a given LLR threshold. We benchmarked CLIC separately using the CORUM database (310 gene sets, 3139 total test genes), the KEGG database (89 gene sets, 2457 total test genes), and the GO cellular component database (511 gene sets, 10277 total test genes). We created receiver-operator curves (ROC) by varying the LLR threshold, and calculating the mean precision (% of gene predictions > LLR threshold that are test genes) and recall (% of test genes predicted at the LLR threshold). We also performed LOOCV using average Pearson correlation with all genes in the input gene set (AvCorr). Human gene sets were mapped to mouse genes using best-bidirectional hits (BlastP expect <1e-3) and analyses were run using the mouse mRNA compendium from platform Mouse430_v2.

#### Immunofluorescence

For immunostaining, HeLa cells were transfected with pDsRed2-Mito (Clontech) using lipofectamine 2000 (Invitrogen) and grown on glass coverslips for 2 days before fixation in 4% paraformaldehyde, blocking in Abdil (PBS, 0.2% Triton X-100, 3% BSA (Sigma)) and immunolabeling with anti-C7orf55 (Abcam ab188310). Secondary donkey anti-rabbit IgG A488-conjugated antibodies (Abcam ab150073) were used and cells were labeled with Hoescht 33342 (Sigma). Microscopy was performed using a Zeiss LSM700 confocal microscope.

### *C*7orf55 gene disruption and reintroduction

All cell culture was performed using DMEM containing 110mg/l pyruvate and 50mg/l uridine (Life Technologies). Three independent CRISPR guides targeting C7orf55 gene (sgRNA1: CACAAGGTACCGATAGGCCG; sgRNA2: CCGACCCTATCGCGACACCG; sgRNA3: GCTCCCGTACCCGATGTGCA) from were cloned in pLentiCRISPR V2 (Addgene) and lentiviruses were produced in HEK 293T cells according to Addgene‘s instruction. K562 cells were infected with all three viruses together and selected with 0.2μg/ml puromycin (Life Technologies) for 2 days. A version of pLentiCRISPR targeting GFP was used as negative control (Addgene). In parallel, a 3xFLAG-tagged CRISPR resistant cDNA of C7orf55 encoding several silent mutations in the CRISPR targeting regions (lower case) was in vitro synthesized (Genewiz) (ATGGCGGCCTTAGGGTCCCCGTCGCACACTTTTCGAGGACTTCTGCGGgaattacgttatctaagtGCGGCCACCGGCcgcccttaccgggatacaGCGgcataccgttatctagttAAGGCTTTCCGTGCACATCGGGTCACCAGTGAAAAGTTGTGCAGAGCCCAACATGAGCTTCATTTCCAAGCTGCCACCTATCTCTGCCTCCTGCGTAGCATCCGGAAACATGTGGCCCTACATCAGGAATTTCATGGCAAGGGTGAGCGCTCGGTGGAGGAGTCTGCTGGCTTGGTGGGTCTCAAGTTGCCCCATCAGCCTGGAGGGAAGGGCTGGGAGCCA). The cDNA was subcloned in pWPI-Neo (Addgene) and lentiviruses were produced as above. After puromycin withdrawal, cells were infected with these lentiviruses and selected 24h after infection in 0.5 mg/ml G418 (Life Technologies) for 2 days. Cells were then washed and kept in exponential growth. All experiments were performed 10–20 days post-infection.

### Protein immunoblotting

Cells were lysed in lysis buffer (50 mM Tris/HCl [pH 7.5], 150 mM NaCl, 1 mM MgCl2, 1% NP-40, 3 mM vanadylate RNase complex) and spun for 5min at 2’000g to remove insoluble material. Protein concentration in the supernatant was measured using Bio-Rad DC protein assay before electrophoresis on a 10%-20% polyacrylamide gel (Life Technologies) and transfer on a PVDF membrane (Biorad). All immunoblots were done in 5% non-fat milk powder in TBS + 0.1% Tween-20 using anti-OXPHOS cocktail antibody (Abcam ab110411 –contains antibodies to ATP5A, UQCRC2, SDHB, COXII and NDUFSB8), C7orf55 antibody (Abcam ab188310) and anti-ATP5B antibody (Abcam ab14730).

### Co-Immunoprecipitation and mass spectrometry

A mitochondria-rich fraction was isolated from HEK 293T cells stably expressing C7orf55-FLAG using mechanical cell disruption and differential centrifugation. Cells were scraped and washed twice with PBS before resuspension in MB buffer (210 mM mannitol, 70 mM sucrose, 10 mM HEPES-KOH [pH 7.4], 1 mM EDTA). Cells were then homogenized using a glass homogenizer (Kontes) and centrifuged at 2000g for 5 minutes. The supernatant was further centrifuged at 13,000g for 10min and the mitochondria-rich pellet was saved and washed in MB. The quality of cellular subfractionation was ensured by running an equal amount of proteins from total cells or from the mitochondria-rich fraction and immunoblotting using anti-OXPHOS cocktail antibody (Abcam ab110411 –contains antibodies to ATP5A, UQCRC2, SDHB, COXII and NDUFSB8). For immunoprecipitation the mitochondria-rich fraction was resuspended in lysis buffer containing 50 mM Tris/HCl (pH 7.5), 150 mM NaCl, 1 mM MgCl_2_, 1% NP-40 and 1× protease and phosphatase inhibitor (Cell Signaling Technology). Lysates were added to anti-FLAG M2 magnetic beads (Sigma) and immunoprecipitation was performed overnight. Beads were then extensively washed in lysis buffer and the immunoprecipitated proteins were recovered using FLAG peptide (Sigma) before protein precipitation with TCA. Mass spectrometry analysis was performed at the proteomics facility of the Whitehead Institute (Cambridge, MA). Mitochondria isolated from HEK 293T cells expressing GFP (control 1) or an unrelated mitochondrial FLAG-tagged protein (control 2) were used as control. Relative peptide abundance was quantified with Scaffold using the Top 3 TIC method and proteins of interest were filtered based on their absence in the controls and their mitochondrial localization (37). For ATPAF2-C7orf55 co-immunoprecipitation, a V5-tagged version of ATPAF2 cDNA was obtained from the Broad Institute ORFeome and transfected in the C7orf55-FLAG expressing cells and 2 days later an immunoprecipitation was performed using anti-V5 (Abcam ab9116) or anti-FLAG M2 antibodies (Sigma) using a dynabeads immunoprecipitation kit (Life Technologies).

### Blue-native PAGE electrophoresis

For blue-native PAGE, a mitochondria-rich fraction was isolated from the K562 cells described above and 50μg of mitochondria were resuspended in blue-native loading buffer containing 1% digitonin (Life Technologies) before electrophoresis on a 3 to 12% Native PAGE (Life Technologies) according to the manufacturer’s instruction except that only low coomasie cathode buffer was used. In gel ATPase activity was performed according to [56].

## Supporting information

S1 Text Supporting statistical details of sample correlations. (DOCX)

S1 Table Top pathway predictions for uncharacterized genes (related to Fig 5). (XLSX)

S2 Table Co-expression of Complex V with C7orf55 using different co-expression tools. (XLSX)

S1 Fig Cumulative number of GEO datasets from 2001 to 2015. (EPS)

S2 Fig Systematic performance evaluation of CLIC on Affymetrix Human U133_Plus_2.0 platform. Leave-one-out cross-validation shown for CORUM (A, D), KEGG (B, E) and GO (C, F) gene sets using 1887 human datasets from Affymetrix microarray HG-U133_Plus_2.0 (A-C) Precision-recall curves show results based on CLIC and average correlation (AvCorr) using GNFv3 tissue atlas. (D-F) Recall-rank curves show the recall (sensitivity) of different methods when looking at only top *N* predictions (*N* ranging 10–400). Results are shown for all gene sets, as well as for subsets with different CEM strength *ϕ* cut-offs.
(EPS)

S3 Fig CLIC results on the highest strength gene sets. Expression profiles are shown for the four gene sets with the highest CEM1 strength: KEGG Oxidative Phosphorylation (A), KEGG Ribosome (B), CORUM 55S mitochondrial ribosome (C), and Condensed chromosome kinetochore (D). Heatmaps show the expression profiles in the three datasets with highest weights. Each row shows one gene, each column shows one sample, and the color gradient shows the expression profile *z*-scores across samples in the corresponding dataset. Blue text shows CEM gene names, green text shows CEM+ gene names (top 10 only), and green arrowheads show predictions with recent experimental or human genetic support for functional association with the input set. Red arrowhead indicates evidence for the mouse homolog of C7orf55 which we experimentally validated as relevant for complex V.
We note that CLIC partitions the KEGG Oxidative Phosphorylation gene set (100 genes) into two non-singleton CEMs: CEM1 contains 53 true mitochondrial Oxidative Phosphorylation genes, while CEM2 contains 12 genes encoding the vacuolar ATPase (V-ATPase) that are incorrectly assigned to this KEGG pathway (likely because they share an Enzyme Commission number with the mitochondrial ATP synthase). This example demonstrates the importance of CLIC’s partitioning step that is able to identify the genes do not belong to the input pathway and eliminate them before the expansion step. CEM1 has strength *ϕ* = 749, and its expansion list contains 286 genes. Green arrows highlight CEM+ genes that are known to be associated with oxidative phosphorylation process. In particular, the top two CEM+ genes, Cox6c and Atp5k, are true members of oxidative phosphorylation process but are missing from the input gene set due to the gene set annotation error.
(EPS)

S4 Fig Kernel and normal fits of background distributions for datasets with good quality (A, B) and bad quality (C, D).
(EPS)
