# Supplementary material for: CLIC, a tool for expanding biological pathways based on co-expression across thousands of datasets
Source: PLoS Comput Biol. 2017 Jul 18;13(7):e1005653. doi: 10.1371/journal.pcbi.1005653 (PMC5546725; doi:10.1371/journal.pcbi.1005653)
Supplement: S1 Text — (DOCX) [file pcbi.1005653.s001.docx]

**Supplemental Materials to “CLIC, a Tool for …”**

Here we present a detailed analysis of sample correlations, which have been used as a summary of the information revealed by each microarray experiment in our paper, and also the main subjects of our Bayesian model. The analysis provides a reasonably strong theoretical support for modeling the Fisher Z-transformed sample correlations by mixture-Gaussian distributions and the use of Normal-Normal hierarchical models for pairwise correlations among genes in a module in different datasets.

To illustrate whether the Gaussian assumption makes sense, we simulated the following scenarios: assume that $X=(x_{1}$, … $x_{k}$) (*k* maybe in the hundreds) follows the joint Gaussian with mean zero and covariance matrix D, whose diagonal elements are all 1's and off-diagonal elements all equaling to r. Then we simulated multiple observations of $X$, say $X_{1},\ldots, X_{n}$ (*n* in the range of 30~50), which is used to estimate the empirical correlation matrix $\hat{R}$. Each entry $\hat{r}_{ij}$ is transformed to $z_{ij}=\log\frac{1+\hat{r}_{ij}}{1-\hat{r}_{ij}}$. We note that the q-q plot for these Z’s is very straight – almost perfectly normal (in the Supplemental Material). In this case, we note that we can characterize each normal random variable $x_{j}$ as having a common random effect, and an individual effect:

$$x_{j}={\sqrt{r}z}_{0}+\sqrt{1-r}z_{j}$$

where $z_{0}, z_{1},\ldots,z_{k}$ are iid N(01) random variables. Thus, we have

$$E\left( x_{i}x_{j}^{2}x_{k} \right)=r^{2}E\left( z_{0}^{4} \right)+r\left( 1-r \right)E\left( z_{0}^{2}z_{j}^{2} \right)=r+2r^{2}, \mathrm{for} i\neq j\neq k;$$

$$E\left( x_{i}x_{j}x_{k}x_{l} \right)=r^{2}E\left( z_{0}^{4} \right)=3r^{2}, \mathrm{for} i\neq j\neq k\neq l.$$

Thus, any two sample covariances (correlations), $\hat{c}_{ij}$ and $\hat{c}_{kl}$, have the common mean, *r*, and are correlated. Specifically, ${cov(\hat{c}}_{ij} \hat{c}_{kl})\approx$ $\frac{r+r^{2}}{n}$ or $\frac{2r^{2}}{n}$, according to whether $(i,j)$ and $(k,l)$ have a common element or not. Furthermore, since ${var(\hat{c}}_{ij})\approx\frac{1+r^{2}}{n}$, we have ${cor(\hat{c}}_{ij} \hat{c}_{kl})\approx\frac{2r^{2}}{1+r^{2}}$ or $\frac{r+r^{2}}{1+r^{2}}$. The sample correlation $\hat{r}_{ij}$’s, however, are less correlated than the sample covariances (their detailed analyses are beyond the scope of this paper), and their Z-transformed version, $\hat{z}_{ij}$’s, are similarly correlated as the $\hat{r}_{ij}$’s. Thus, in this case, they again can be represented (approximately) as sharing a common random-effect, as with the case of $x_{j}$, i.e.,

$$\hat{z}_{ij}\approx{\alpha v}_{0}+\sqrt{1-\alpha^{2}}v_{ij}$$

where $v_{ij}$ are iid N(0,1) random variables and $\alpha$ is close to $r^{2}$ when *n* is relatively large (empirical observation). Thus, an effect of having correlated $\hat{z}_{ij}$’s is that using them to estimate the population properties (such as mean and variance) becomes less accurate than that using independent ones. But as we show below, this does not affect much when using mixture models to cluster members with similar pair-wise correlations.

Below we showed the qq-plot for an empirical study in which the common true correlation is $r=0.6$; dimension p=500; and sample size n=50, 100, or 10,000. That is, we used 50, 100, or 10,000 multivariate Gaussian observations to estimate the sample correlation matrices, respectively. The number estimated correlations is $\frac{p\left( p-1 \right)}{2}=124,750$. Thus, the plots in the left box below are based on 124,750 correlations. Since they are correlated, their means can be quite different from the theoretical mean 0.693 even when the average is taken with 124,750 numbers, which fits our above random-effect formulation quite well.


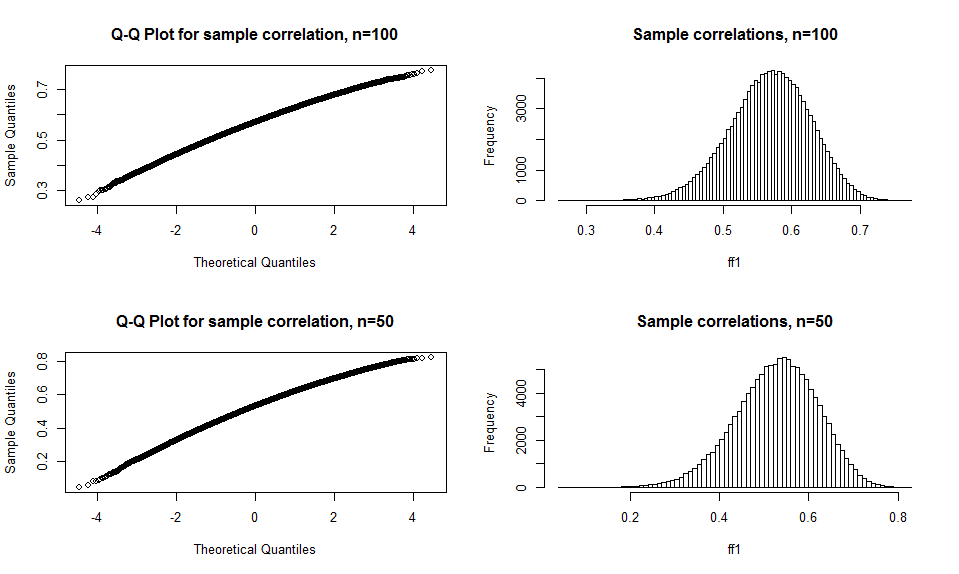

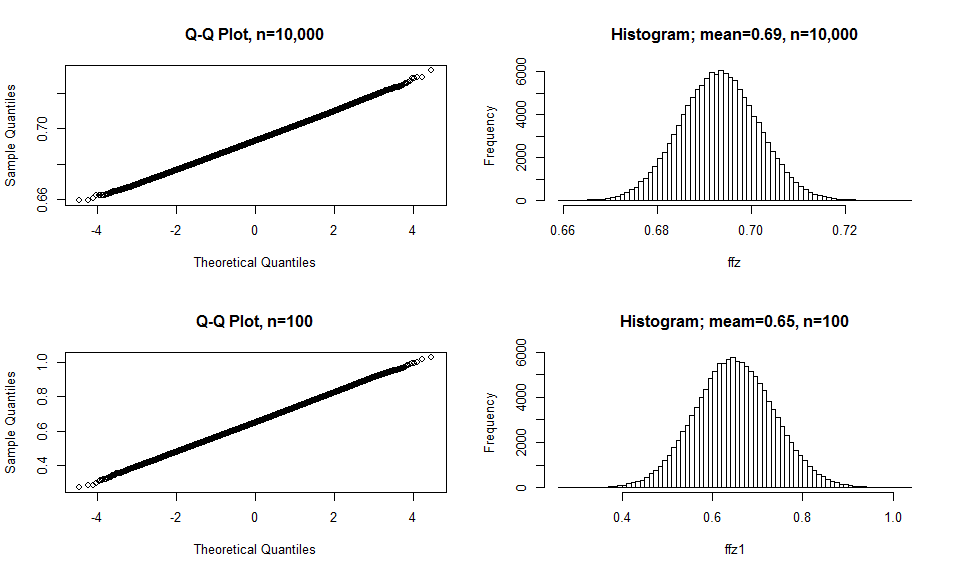


The figures in the right box shows that when n=100 or 50, the untransformed sample correlations are skewed towards left, not exactly like samples from Gaussian distributions:

We have also conducted simulation experiments to study how the sample correlations are correlated in the sense of repeated sampling. For example, with n=50, we did 200 independent replications, and investigated how ${cor(\hat{r}}_{ij} {,\hat{r}}_{kl})$ behave. We observe that when $i\neq j\neq k\neq l$, the correlations centered around 0.3, and otherwise they centered around 0.45.

Figure below shows the box plots of the distributions of the Z-transformed sample correlations in 100 independent replications. Each box plot corresponds to $300\times\frac{299}{2}=44950$ Z-transformed sample correlations computed from 50 observations of 300-dimensional Gaussian with constant pairwise correlations at 0.6. It is seen that the means of these Z-transformed values dance with quite significant variations around the population mean 0.693, which is the z-transformation of the true correlation 0.6. As explained in our earlier theoretical analysis, these large variations are due to the near-constant inter-correlations among any pair of sample correlations. The posited model in CLIC algorithm is consistent with this observation by assuming that the genes in a gene module has its Z-transformed pairwise correlations following a normal distribution with an experiment-specific mean, and these experiment-specific means follow a hierarchical Gaussian model. Thus, the following figure can be viewed as an idealized version of the model: the 100 independent replicates can be viewed as 100 ideal “datasets”, and each “dataset” consists of 50 “samples” and is summarized by a correlation matrix for 300 genes. The Normal-Normal hierarchical model of CLIC appears to be a very good summary/fit of the observations demonstrated by the 100 boxplots in the figure below.


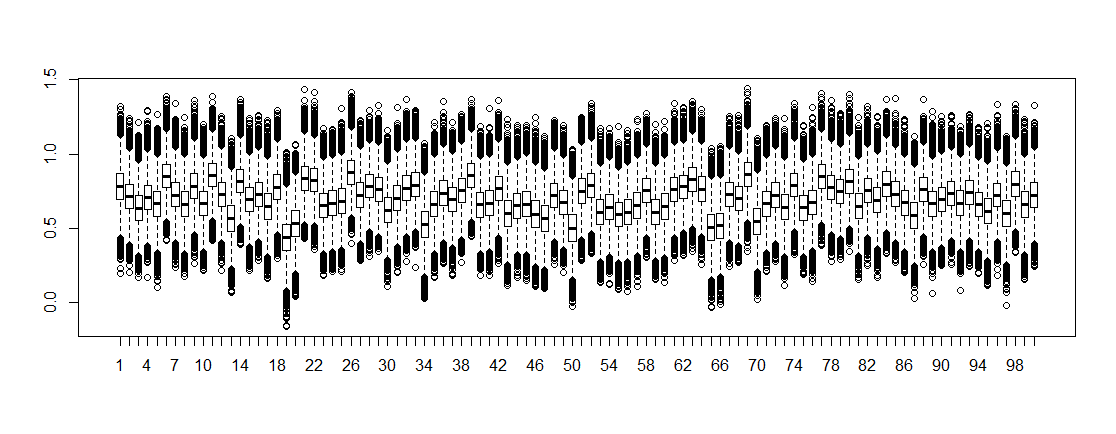


Similar to the computation in Hu and Zhao (2016), we can also model that genes within each module have a common high correlation $r_{1}$ (or $r_{2})$ , but genes between modules have a lower common correlation $r_{12}$. We therefore further simulated a case with p=300, n=50, but the covariance matrix is made from three parts with the first 50 dimensions having a constant correlation$r_{1}=0.7$, and the next 100 dimensions with constant correlation $r_{2}=0.4$, and all remaining 150-dimensional vector simulated from using a random matrix with eigenvalues generated from Unif(0,10) (using the R-function “Posdef”) and then normalized into a correlation matrix. All other cross-term entries were set at zero. Figure below indicates that the z-transformed correlations can be fitted very well by a 3-component mixture Gaussian, which is reasonably good for our purposes. Right figure compares the nonparametric density estimate from using the sample correlations with the mixture model fitting, and we see almost no differences, indicating that the Gaussian model-based clustering in our paper can indeed cluster members with similar sample correlations together.


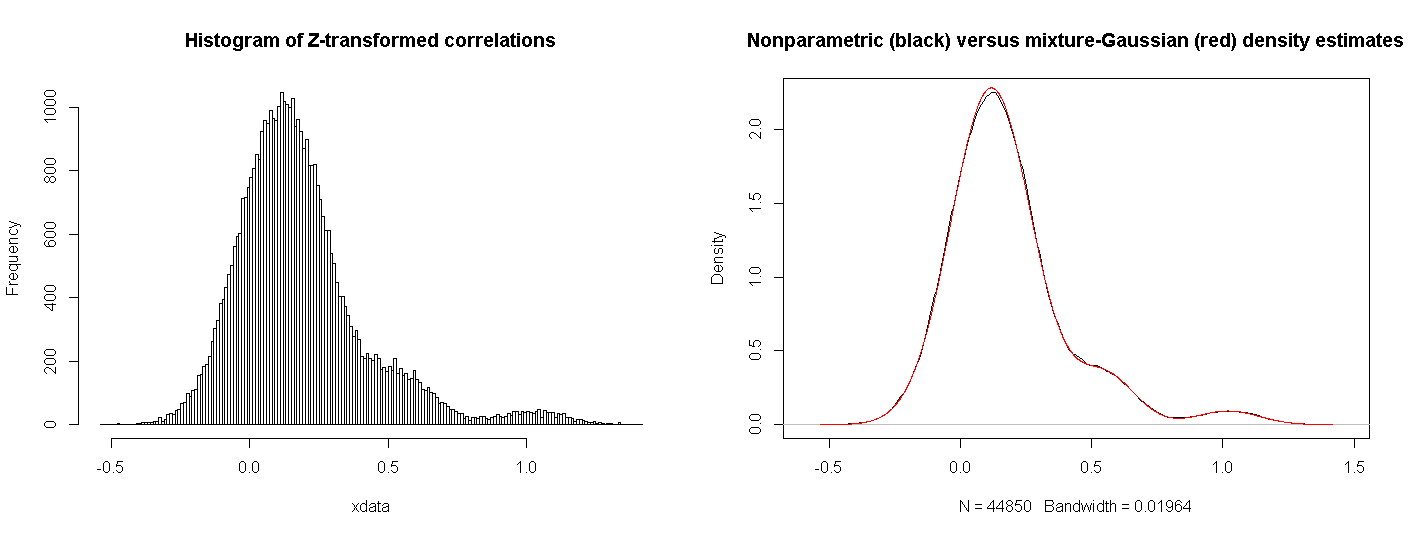


The correlations among the sample correlations can possibly weaken the clustering of genes that share similar correlations, but should not affect the results significantly.

We note that we designed our simulation to mimic the correlation structures present in our real data. That is, we did not arbitrarily construct correlated z’s and pool them together. Instead we simulated exactly the same type of data as our real data: we simulated multiple multivariate Gaussian data sets under the same covariance matrix, computed their sample correlations and z-transformations, and pooled the z’s together. We empirically showed that the conditional independence structure fits these data well.
